# Language and context in cyberbullying research: bibliometric mapping and conceptual integration of lexical, pragmatic, and media-psychological perspectives (2010–2025)

**DOI:** 10.3389/fpsyg.2026.1963458

**Published:** 2026-10-21

**Authors:** Shuangquan Deng, Azianura Hani Shaari, Azahah Abu Hassan Sha’ari, Isma Noornisa Ismail

**Affiliations:** 1 Center for Research in Language and Linguistics, Faculty of Social Sciences and Humanities, The National University of Malaysia, Bangi, Selangor, Malaysia; 2 Center for Research in Psychology and Human Well-Being, Faculty of Social Sciences and Humanities, The National University of Malaysia, Bangi, Selangor, Malaysia; 3 Academy of Language Studies, Universiti Teknologi MARA Cawangan Pulau Pinang, Pulau Pinang, Malaysia

**Keywords:** bibliometrics, cyberbullying, lexical semantics, media psychology, pragmatics

## Abstract

Cyberbullying is a media-psychological phenomenon enacted through language, interpreted within relationships, and conditioned by platform affordances. Yet linguistic and pragmatic research remains fragmented across psychology, communication, media studies, pragmatics, and computational detection. This bibliometric review maps the development and intellectual structure of this research from 2010 to 2025 using 345 English-language records from the Web of Science Core Collection. Annual output increased from 1 publication in 2010 to 51 in 2025, and 218 records (63.2%) were published after 2020. The mapping identified prominent psychosocial and computational knowledge structures, while explicitly pragmatic constructs remained comparatively less consolidated in high-frequency keyword and burst patterns. To interpret these mapped patterns, the second half of the review is organized through four non-mutually exclusive analytical lenses: psychosocial victimization, lexical-semantic harm, pragmatics and discourse, and computational detection. Drawing on the bibliometric findings and theory-informed interpretation, we propose a conceptual language-to-harm framework linking platform affordances, linguistic cues, pragmatic interpretation, psychological appraisal, behavioral and social responses, and cumulative harm. The framework is presented as a theory-informed synthesis and future research model rather than as a causal structure empirically validated by the bibliometric data.

## 1 Introduction

Cyberbullying is commonly defined as intentional and repeated aggression conducted through digital technologies against a person who has difficulty defending themselves, although repetition and power imbalance may be produced differently online than in face-to-face settings (Kasturiratna et al., 2025; Barlett et al., 2024; Lee et al., 2026; Kowalski et al., 2014). A single post can be copied, searched, and encountered again long after it was written, and this persistence—together with its public visibility and the participation of an audience—allows a message to acquire a cumulative force that a spoken insult rarely has. Cyberbullying is, at once, a behavioral, relational, linguistic, and media-psychological phenomenon, and treating it as only one of these tends to lose sight of the others.

Associations between cyberbullying and moral disengagement, hostile attribution, anxiety, depression, and reduced well-being are by now well established (Kowalski et al., 2014; Lo Cricchio et al., 2021; Zhao and Yu, 2021), and more recent samples continue to find the same link between moral disengagement and both cybervictimization and cyberaggression (Dominguez-Vergara et al., 2023). Bandura’s social cognitive theory frames this as a matter of reciprocal influence between personal factors, behavior, and the mediated environment rather than a one-way effect (Bandura, 2001). Moral disengagement theory, drawn from the same tradition, describes how aggressors neutralize their own self-sanctions—through moral justification, euphemistic labeling, diffusion of responsibility, dehumanization, victim blaming (Bandura, 1999). In networked environments this diffusion of responsibility takes on a particular shape: an attack can be sustained by dozens of commenters, each contributing only a fragment, none of whom may feel individually responsible for the whole. The General Aggression Model adds an account of what triggers this in the moment, proposing that situational inputs and person factors shape cognition, affect, and arousal before any appraisal or action follows (Anderson and Bushman, 2002). A provocative post, a hostile label, an audience that responds approvingly—each can function as exactly this kind of situational cue, altering aggressive scripts and the choices that follow from them.

What media psychology contributes is an explanation of why the same words can have such different effects depending on the platform, the user, and the relationship involved. Suler’s (2004) online disinhibition account links anonymity, invisibility, asynchronicity, and the absence of authority cues to a lowering of behavioral restraint, and Wang et al.’s (2024) systematic review substantiates the claim, finding these platform properties consistently associated with adolescent cyberbullying perpetration. Anonymity and invisibility have also been shown, experimentally, to intensify toxic disinhibition, and more recent work traces this same mechanism through trait anger and revenge motivation as specific predictors of perpetration (Ding et al., 2025). Walther’s (1996) account of computer-mediated communication complicates the disinhibition story somewhat: online interaction is not simply cue-deficient, since users adapt to the verbal and relational cues that are available, form impressions from them, and at times experience more intense interpersonal dynamics as a result. The Differential Susceptibility to Media Effects Model makes a related point in more general terms, treating media effects as conditional on a user’s dispositions, developmental stage, and social context rather than uniform across users (Valkenburg and Peter, 2013); the model has since been applied directly to cyberbullying as a predictor of problematic social media use in a nationally representative adolescent sample (van Duin et al., 2021). Tie strength appears to matter too—on Chinese platforms, how closely users are connected moderates whether social-media use translates into cyberbullying involvement at all (Ni et al., 2025).

Much of this is ultimately carried by language. Lexical selection categorizes a target and activates a stereotype; metaphor can dehumanize without any single word doing so on its own; address forms position speaker and target relative to each other; repetition normalizes what would otherwise register as hostile. Impoliteness scholarship makes the underlying point directly: offense is produced through situated evaluations of language and social relations, not through some fixed inventory of offensive forms (Culpeper, 2011), and recent cyberpragmatic work continues to bear this out in social-network-service contexts specifically (Rhee, 2023). The same string of words can function as an attack, a quotation, a reclaimed identity expression, a parody, or counterspeech, and which one it is depends on speaker commitment, target, sequence, audience, and local norms—none of which is recoverable from the words alone. Online aggression also works through insinuation, presupposition, sarcasm, exclusion, dog-whistles, and coordinated visibility, often without a single profane term appearing anywhere (Marwick and Caplan, 2018; Dynel and Poppi, 2019; Hardaker, 2010; Zhong et al., 2022). Pragmatic interpretation sits between linguistic form and psychological appraisal; without it, the form alone tells you very little.

A separate, largely computational literature has treated cyberbullying, hate speech, toxicity, and offensive language as text-classification problems, with methods moving from commonsense reasoning and engineered lexical features toward convolutional networks, contextual embeddings, attention mechanisms, and ensembles (Salawu et al., 2020; Chia et al., 2021; Lu et al., 2020; Dewani et al., 2021; Iwendi et al., 2023). Scalability has improved considerably but so has a construct-validity problem that scalability does not resolve. Cyberbullying is ordinarily relational and unfolds over time, while most training data consist of isolated, decontextualized sentences. A model can therefore detect offensive wording without ever confirming repetition, power asymmetry, target vulnerability, hostile intent, or cumulative harm—and, in the other direction, can miss coded or indirect aggression whenever it relies on salient lexical cues that simply are not present. Work on abusive-language taxonomies, unintended identity bias, and counterspeech has shown what this costs in practice: label design and context determine who is protected and whose ordinary language gets mistakenly flagged as abuse (Waseem et al., 2017; Borkan et al., 2019; Sap et al., 2019).

Cyberbullying is treated in this review as the focal construct rather than as a synonym for all forms of online aggression. For terminological consistency, cyberbullying refers to target-directed harmful online conduct in which repetition or persistence, power asymmetry, target vulnerability, or cumulative exposure may be relevant to interpretation. Online aggression is used as the broader umbrella term for digitally mediated aggressive conduct and does not, by itself, imply all defining features of cyberbullying. Cyber aggression refers to aggressive behavior enacted through digital communication and may include single or repeated acts; and verbal aggression and verbal abuse refer primarily to harmful linguistic conduct at the message or discourse level. These neighboring constructs may overlap with cyberbullying but do not necessarily satisfy all of its defining conditions. These terms are therefore retained where required by the source literature or retrieval strategy, but they are not used interchangeably in the analysis.

Existing reviews have already synthesized cyberbullying measurement, correlates, mental-health outcomes, and automated detection (Kowalski et al., 2014; Salawu et al., 2020; Bansal et al., 2024), and bibliometric work has traced the field’s growth and geographic spread. What remains less clearly understood, however, is how linguistic and pragmatic representations are positioned in relation to the psychological processes and outcomes that dominate the broader literature. Lexical and computational studies often focus on identifying or classifying harmful language, whereas psychosocial research examines constructs such as online disinhibition, moral disengagement, victimization, appraisal, and psychological harm. These strands have frequently developed in parallel, leaving limited integration of how linguistic forms acquire pragmatic meaning and how such interpretations may relate conceptually to psychological appraisal, social response, and harm. This limited integration constitutes the central research gap addressed by the present review.

The present review therefore combines performance analysis, science mapping, and theory-informed interpretation of the mapped structures to examine the development of lexical and pragmatic cyberbullying research from 2010 to 2025. Taken together, these theories explain different components of cyberbullying rather than functioning as competing explanations: media affordances may reduce restraint, cognitive mechanisms may legitimize aggression, linguistic and relational cues shape interpretation, and individual susceptibility conditions psychological and behavioral responses.

For analytical clarity, four cross-disciplinary terms are used operationally throughout the review. Lexical refers to word- and phrase-level forms and semantic representations through which harmful evaluation or targeting is encoded, including insults, slurs, labels, sentiment-bearing expressions, collocations, and related lexical-semantic features. Pragmatic refers to context-dependent meaning and communicative force that cannot be determined from lexical form alone, including stance, irony, sarcasm, implicature, politeness or impoliteness, interactional sequence, and audience uptake. Media-psychological refers to psychological processes and outcomes interpreted in relation to mediated conditions, including online disinhibition, moral disengagement, appraisal, victimization, mental-health outcomes, and platform affordances. Computational detection refers to the automated identification or classification of harmful online communication using natural-language-processing, machine-learning, deep-learning, feature-extraction, embedding, or related computational methods. These categories are analytically distinct but may overlap within the same publication.

The research questions are as follows: (RQ1) How have publication output, sources, authorship, citations, and national participation developed? (RQ2) How are linguistic, psychosocial, media, and computational approaches organized within collaboration, keyword, and co-citation structures? (RQ3) Which lexical, pragmatic, psychosocial, and computational research areas are visible in the bibliometric structures, and how have their prominence and relationships changed over time? (RQ4) How are linguistic and pragmatic perspectives conceptually positioned in relation to psychological processes and outcomes within the mapped literature? (RQ5) What conceptual and methodological priorities for future cyberbullying research emerge from the bibliometric findings and theory-informed synthesis?

## 2 Materials and methods

### 2.1 Review design and reporting

This study employed a bibliometric review based on a systematic and reproducible search and corpus-construction procedure, integrating performance analysis, science mapping, and theory-informed interpretation of the resulting bibliometric structures (Zupic and Čater, 2015). The study was not designed as a conventional systematic review involving study-level evidence synthesis, full-text eligibility assessment, or risk-of-bias appraisal. Instead, the unit of analysis was the bibliographic record, and the systematic search procedure was used to construct a clearly bounded corpus for bibliometric mapping.

The identification and corpus-construction process was reported with reference to the transparency principles of PRISMA 2020 (Page et al., 2021). In the present study, PRISMA was used to document database identification, predefined database restrictions, duplicate checking, metadata verification, and final corpus construction; it was not intended to imply a conventional title/abstract/full-text screening process or study-level evidence synthesis. Although the manuscript is submitted under Frontiers’ Systematic Review article type, its methodological design is bibliometric rather than a conventional systematic review of study-level evidence. No protocol was prospectively registered.

The bibliometric and interpretive components were integrated sequentially rather than treated as independent analyses. Performance analysis and science mapping first established the empirical bibliometric structure of the corpus, including publication patterns, keyword configurations, co-citation communities, temporal signals, and citation relationships. These mapped structures then provided the evidential basis for theory-informed interpretation. The interpretive stage contextualized, rather than altered, the bibliometric findings by examining bibliometrically anchored representative titles, abstracts, keywords, and cited works in relation to the predefined analytical lenses and relevant media-psychological theories. Accordingly, bibliometric patterns are reported as empirical findings, whereas psychological mechanisms and cross-domain relationships are treated as theory-informed interpretations or conceptual propositions requiring future empirical testing.

Operational identification applied the definitions above to bibliographic metadata and bibliometrically anchored representative records, using dominant conceptual and methodological content rather than the occurrence of a single keyword. Records could contribute to more than one analytical lens; the detailed selection and lens-assignment procedure is specified in Section 2.4.

Because the units of analysis were published bibliographic records rather than human participants, institutional ethics review was not required.

### 2.2 Data source, search strategy, and corpus construction

The source file was exported from the Web of Science Core Collection on 30 May 2026 and covered the predefined publication window of 2010–2025. The query required every record to satisfy two conceptual blocks simultaneously: a target-phenomenon block containing cyberbullying and adjacent online-aggression terminology, and a linguistic-dimension block containing lexical, discourse, pragmatic, and verbal-aggression terminology (Table 1). SSCI, A&HCI, SCI-EXPANDED, and ESCI were searched, with English language and specified document types applied as database filters.

**Table 1:** Search strategy and data extraction parameters in web of science.

Category/field	Search parameters/query strings
Database	Web of Science Core Collection: SSCI, A&HCI, SCI-EXPANDED, and ESCI
Concept block 1: target phenomenon	TS = (cyberbull* OR “cyber bully*” OR “online bullying” OR “online harassment” OR “cyber harassment” OR “cyber-harassment” OR “cyber aggress*” OR cyberaggress* OR “digital bullying” OR “internet bullying”)
Concept block 2: linguistic dimensions	TS = (lexic* OR lexicon* OR vocabular* OR “word choice” OR “word use” OR “word usage” OR “linguistic feature*” OR “linguistic pattern*” OR “language use” OR “language pattern*” OR discourse* OR pragmat* OR “speech act*” OR impoliteness OR politeness OR implicature* OR presupposition* OR sarcas* OR iron* OR insult* OR slur* OR profan* OR metaphor* OR euphemis* OR dysphemis* OR stance OR “evaluative language” OR “identity construction” OR “verbal aggression” OR “verbal abuse”)
Boolean operator	Concept block 1 AND Concept block 2
Publication years	2010–2025
Document types	Article, Review Article, Data Paper; Book Chapter excluded
Language	English
Export date and format	30 May 2026; full plain-text bibliographic records

Because the second conceptual block required lexical, pragmatic, discourse, or verbal-aggression terminology, the resulting corpus preferentially captures cyberbullying research in which linguistic dimensions are explicitly represented in titles, abstracts, or indexed keywords. Relevant cyberbullying studies that do not use these terms may therefore be underrepresented or absent from the retrieved dataset. Accordingly, the mapped structures and their interpretation should be understood as characteristics of a linguistically focused, search-defined Web of Science corpus rather than as an exhaustive representation of the entire cyberbullying literature.

Web of Science Core Collection was used as a single, internally consistent bibliographic source because the analyses required standardized cited-reference, affiliation, source, and keyword metadata across performance and network analyses. The use of a single database also avoided inconsistencies that can arise when heterogeneous citation metadata from different indexing platforms are merged. This choice may nevertheless underrepresent publications indexed exclusively in other databases, particularly linguistics, communication, computer-science, or regional outlets; the implications of this database boundary are considered in Section 4.7.

As defined in the Introduction, adjacent online-aggression terms, including online harassment, cyber aggression, and verbal abuse, were retained to broaden retrieval of linguistically and computationally relevant scholarship. Their inclusion served a corpus-retrieval function and did not imply conceptual equivalence with cyberbullying.

The final Web of Science export contained 345 records: 329 articles, 14 reviews, and two data papers. DOI matching identified no duplicates. Titles, abstracts, keywords, document type, language, and publication year were checked only to verify conformity with the predefined search and database conditions and to identify obvious indexing errors. This verification step did not constitute conventional study-level title/abstract screening or full-text eligibility assessment, and no additional topical exclusion criteria were applied after export. Consequently, all 345 retrieved records satisfying the predefined corpus conditions were retained as the primary bibliometric corpus.

### 2.3 Bibliometric and network analysis

Bibliographic data were converted and analyzed in R 4.6.1 using bibliometrix 5.4.1 (Aria and Cuccurullo, 2017). Performance analysis covered annual scientific production, sources, authors, countries, author keywords, Keywords Plus, and global citations. Country counts were derived from author-affiliation fields using full counting; a multinational paper therefore contributed once to every represented country. Citation counts were those stored in the Web of Science export and are time sensitive.

Science mapping included country collaboration, author-keyword co-occurrence, reference co-citation, keyword temporal-overlay, burst-detection, and journal dual-map analyses. VOSviewer 1.6.18 was used for network construction, association-strength normalization, clustering, and visualization (van Eck and Waltman, 2010). The country collaboration network retained countries with at least one publication. The author-keyword co-occurrence network retained terms occurring at least 10 times, whereas the keyword temporal-overlay map retained terms occurring at least five times. The reference co-citation network retained cited references occurring at least 10 times. CiteSpace 6.4. R1 used one-year time slices and g-index node selection with k = 25; Pathfinder and pruning of merged networks were applied consistently. CiteSpace supported keyword-burst detection, co-citation interpretation, and the journal dual-map overlay (Chen, 2006), and the 25 strongest keyword bursts were retained for presentation. Thresholds were specified before substantive interpretation to balance information retention with network readability and to reduce the influence of isolated, low-frequency nodes. The 10-occurrence thresholds for the author-keyword network focused the displayed structure on recurrent topical signals, while the five-occurrence threshold for the temporal-overlay map preserved greater temporal detail; the co-citation threshold of 10 focused interpretation on recurrent intellectual linkages rather than incidental citations. Because node-selection parameters and corpus definition may influence network structure and cluster visibility, robustness was further evaluated through the sensitivity analysis described below. Absence from a displayed network therefore means that a term, country, or reference did not satisfy the stated display threshold and does not establish substantive absence from the corpus.

As a supplementary validation of pragmatic-term visibility, record-level counts were calculated across title (TI), abstract (AB), author keywords (DE), and Keywords Plus (ID) for the following case-insensitive patterns specified in the editorial request: pragmat*, implicature*, “speech act*,” stance, sarcas*, iron*, politeness, and impoliteness. Each record was counted once per field regardless of repeated within-field occurrences. The resulting cross-field counts are reported in Supplementary Table S2.

### 2.4 Theory-informed and media-psychological interpretation

Theory-informed interpretation followed, and was anchored to, the empirical bibliometric mapping rather than constituting an independent qualitative thematic analysis. The procedure comprised three linked steps. First, author keywords, Keywords Plus, co-citation structures, and temporal or burst signals were examined to identify recurrent and structurally salient patterns. Second, representative records were purposively selected from the mapped structures for interpretive illustration rather than for frequency estimation. Priority was given to records that were bibliometrically prominent within a major structure, directly illustrated a salient lexical-semantic, pragmatic/discourse, psychosocial, or computational pattern, or helped clarify a relationship between mapped research areas. Titles, abstracts, keywords, and cited works from these records were then interpreted through four overlapping theory-informed analytical lenses: psychosocial victimization, lexical-semantic representation of harm, pragmatics and discourse context, and computational detection. Third, interpretations were developed by triangulating the mapped cluster and keyword patterns, temporal signals, and the content of these illustrative records with the relevant media-psychological theories, with particular attention to potential relationships among media affordances, linguistic cues, pragmatic interpretation, psychological appraisal, social response, and harm. The four lenses were used to organize interpretation rather than to classify every publication into mutually exclusive categories. No attempt was made to conduct formal full-text thematic coding of all records, estimate the prevalence of the four lenses, or treat the illustrative records as a statistically representative sample. Automated term matching supported navigation within the corpus, but the analytical-lens labels and conceptual interpretations remained grounded in the mapped bibliometric structures and the purposively selected records.

A total of 15 unique illustrative records were purposively selected from the mapped 345-record corpus for theory-informed interpretation, yielding 16 lens assignments because one record was used under two non-mutually exclusive lenses. The lens-specific distribution was psychosocial victimization (*n* = 1), lexical-semantic harm (*n* = 6), pragmatics and discourse (*n* = 3), and computational detection (*n* = 6). The complete bibliographic identities, Web of Science accession numbers, lens assignments, and reasons for illustrative selection are reported in Supplementary Table S3. External theory, meta-analysis, review, and classifier-bias sources used to contextualize or interpret the mapped records were not counted as illustrative corpus records.

Assignment of a mapped cluster or representative record to an analytical lens was based on its dominant conceptual and methodological content rather than on the presence of a single keyword. Psychosocial victimization was used when the mapped content centered on victimization, perpetration, mental health, developmental, cognitive, affective, or behavioral processes. Lexical-semantic harm was used when the primary emphasis concerned harmful lexical representation, offensive language, slurs, sentiment, semantic features, or identity-based linguistic cues. Pragmatics and discourse were applied when interpretation depended on context, stance, irony, sarcasm, implicature, interactional sequence, audience uptake, or discourse organization. Computational detection was used when the principal focus was automated classification, machine learning, deep learning, feature extraction, embeddings, or natural-language-processing methods. Because these areas overlap, the four lenses were treated as non-mutually exclusive interpretive categories.

The psychological interpretation drew on complementary theories with distinct explanatory roles. Online disinhibition described how mediated conditions can reduce restraint (Suler, 2004); moral disengagement described how self-sanctions can be neutralized (Bandura, 1999); the General Aggression Model described cognition, affect, arousal, appraisal, and action pathways (Anderson and Bushman, 2002); computer-mediated communication theory described adaptation to verbal and relational cues (Walther, 1996); and the Differential Susceptibility to Media Effects Model highlighted conditional effects across users and social contexts (Valkenburg and Peter, 2013). Psychological mechanisms were interpreted with reference to these established theories and were not estimated as causal effects from bibliographic metadata.

### 2.5 Sensitivity and robustness analysis

To assess whether the principal bibliometric structure depended on the broad inclusion of adjacent online-aggression terminology, a sensitivity analysis was conducted using a more narrowly defined cyberbullying-focused subcorpus. From the primary corpus of 345 records, 254 records were retained under the narrower corpus definition, representing 73.62% of the original dataset, while 91 records were excluded. The narrower subcorpus was constructed by applying the case-insensitive regular expression `\bcyber-?bully` to four Web of Science fields only: title (TI), abstract (AB), author keywords (DE), and Keywords Plus (ID), including field-continuation lines. A record was retained when the pattern occurred in at least one of these fields; matches occurring only in cited references (CR) or other non-target fields were not counted. The rule captured concatenated and hyphenated cyberbullying forms but intentionally did not capture the spaced form “cyber bullying.” The retained records were structurally validated, with no malformed records or duplicate Web of Science accession identifiers (UTs). The complete list of the 91 excluded records is provided in Supplementary Table S1.

The sensitivity analysis was extended beyond the co-citation clustering to the author-keyword co-occurrence network and keyword-burst analysis. For direct comparison, the 254-record author-keyword network was rerun using the same minimum-occurrence threshold of 10 as the primary 345-record network. The keyword-burst analysis was also rerun for the narrower corpus, retaining the 25 strongest bursts under the same analytical configuration. Co-citation clustering was repeated independently for the primary and narrower corpora using the same analytical settings: a predefined 2010–2025 analytical window, one-year time slices, g-index selection with k = 25, Pathfinder, and pruning of merged networks. Network robustness was assessed by comparing the overall cluster structure across the two corpora and by reporting network size, edge count, density, modularity Q, weighted mean silhouette, and the harmonic mean of Q and S. The comparison focused on the persistence of major intellectual strands rather than requiring identical automatically generated cluster labels across corpora. The corresponding narrower-corpus the co-citation clustering, the author-keyword co-occurence network and keyword burst analysis plot are provided as Supplementary Figures S2S4.

### 2.6 Quality, bias, and reproducibility considerations

A conventional study-level risk-of-bias instrument was not applied because the review did not pool intervention effects, prevalence estimates, or diagnostic accuracy. Instead, quality appraisal focused on corpus-level threats: single-database coverage, English-language restriction, document-type filtering, citation-age advantage, metadata inconsistency, keyword dependence, full counting, and threshold sensitivity. The theory-informed interpretation was not based on formal study-level coding or independent double-coding. Consequently, the four analytical lenses should be regarded as a transparent conceptual interpretation of the bibliometric structures rather than a reliability-tested content analysis. The single-database design may omit relevant publications indexed exclusively elsewhere, and this limitation is particularly relevant to linguistic and pragmatic research distributed across heterogeneous disciplinary outlets. The sensitivity analysis addresses robustness to corpus definition but does not remove database-coverage bias.

## 3 Results

### 3.1 Performance and intellectual structure

#### 3.1.1 Bibliometric corpus and publication growth

The targeted intersectional query yielded 345 records, all of which satisfied the predefined database-level year, language, document-type, and two-block search conditions. DOI checking identified no duplicates, and metadata verification identified no records requiring post-export removal. Accordingly, all 345 records constituted the final bibliometric corpus and entered performance analysis and science mapping (Figure 1). Annual output rose from one publication in 2010 to 51 in 2025, producing 345 documents in total (Figure 2). The first period, 2010–2015, produced 31 records (9.0%). Output expanded during 2016–2019, with 67 records, and then accelerated after 2020. The years 2020–2025 accounted for 247 records (71.6%), while 2021–2025 alone accounted for 218 (63.2%). The trajectory was not perfectly linear—output dipped in 2019 and 2022—but the sustained post-2020 level indicates sustained publication activity within the literature retrieved by the present WoS search strategy rather than a short-lived publication spike. The timing is consistent with increased platform-text availability, rising concern about online harm, and wider adoption of deep-learning methods; these explanations are interpretive and are not tested as causal drivers by the annual-count data.

**Figure 1:**
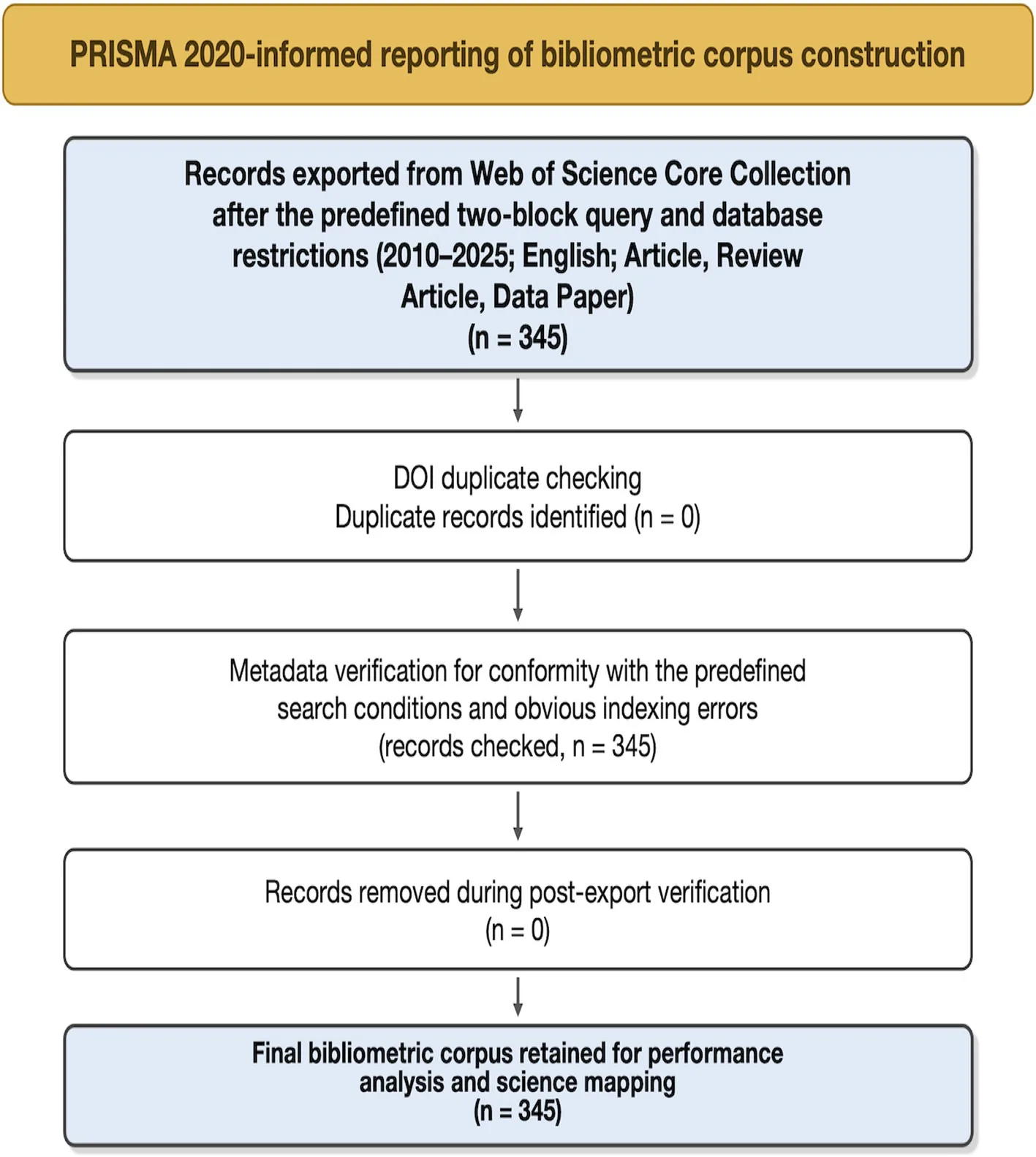
PRISMA 2020-informed flow diagram documenting systematic record identification and bibliometric corpus construction. PRISMA 2020 is used here as a reporting framework for transparent corpus construction; no conventional title/abstract/full-text study-level screening or risk-of-bias appraisal was conducted.

**Figure 2:**
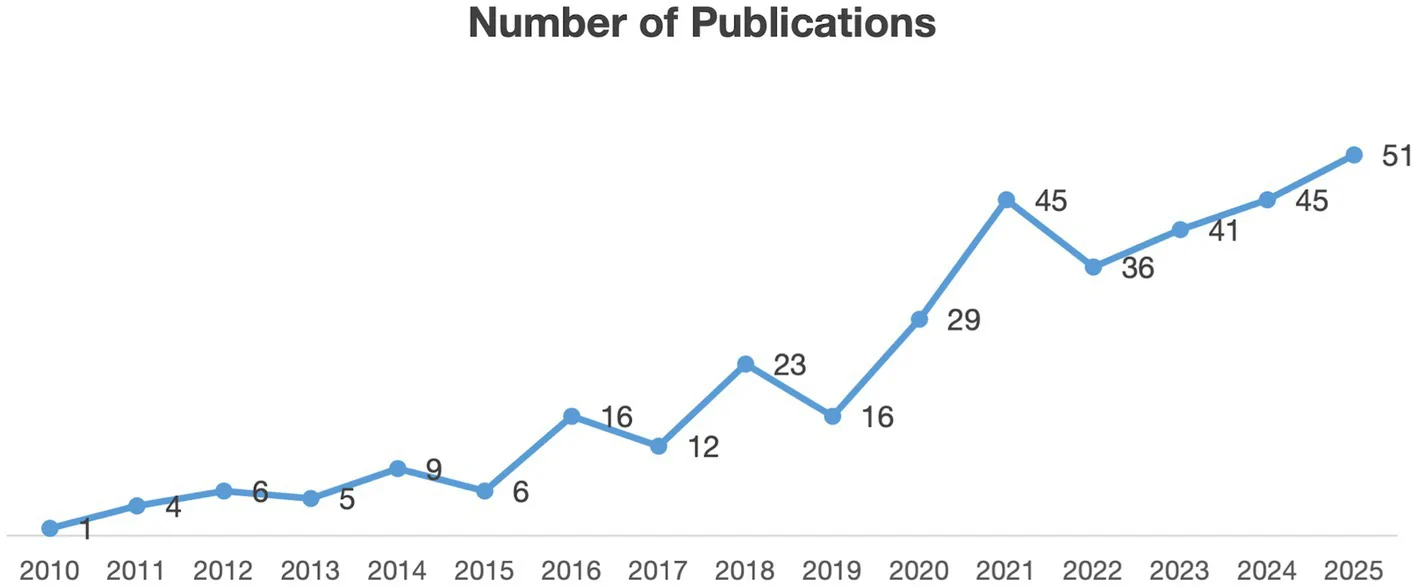
Annual number of publications in the analyzed corpus, 2010–2025. The x-axis represents publication year and the y-axis the number of records published in each year. All records in the final corpus are included; no additional frequency threshold was applied.

#### 3.1.2 Sources, authors, and geographic participation

The 345 publications appeared in 226 sources, demonstrating substantial disciplinary dispersion. Computers in Human Behavior was the most productive outlet (14 documents), followed by Feminist Media Studies and IEEE Access (nine each), and the International Journal of Advanced Computer Science and Applications and International Journal of Environmental Research and Public Health (seven each). The longer source distribution included outlets in communication, psychology, public health, gender studies, law, semiotics, computer science, and artificial intelligence (Figure 3). Within the retrieved WoS corpus, this distribution indicates a cross-disciplinary research space rather than a stable single-discipline specialty.

**Figure 3:**
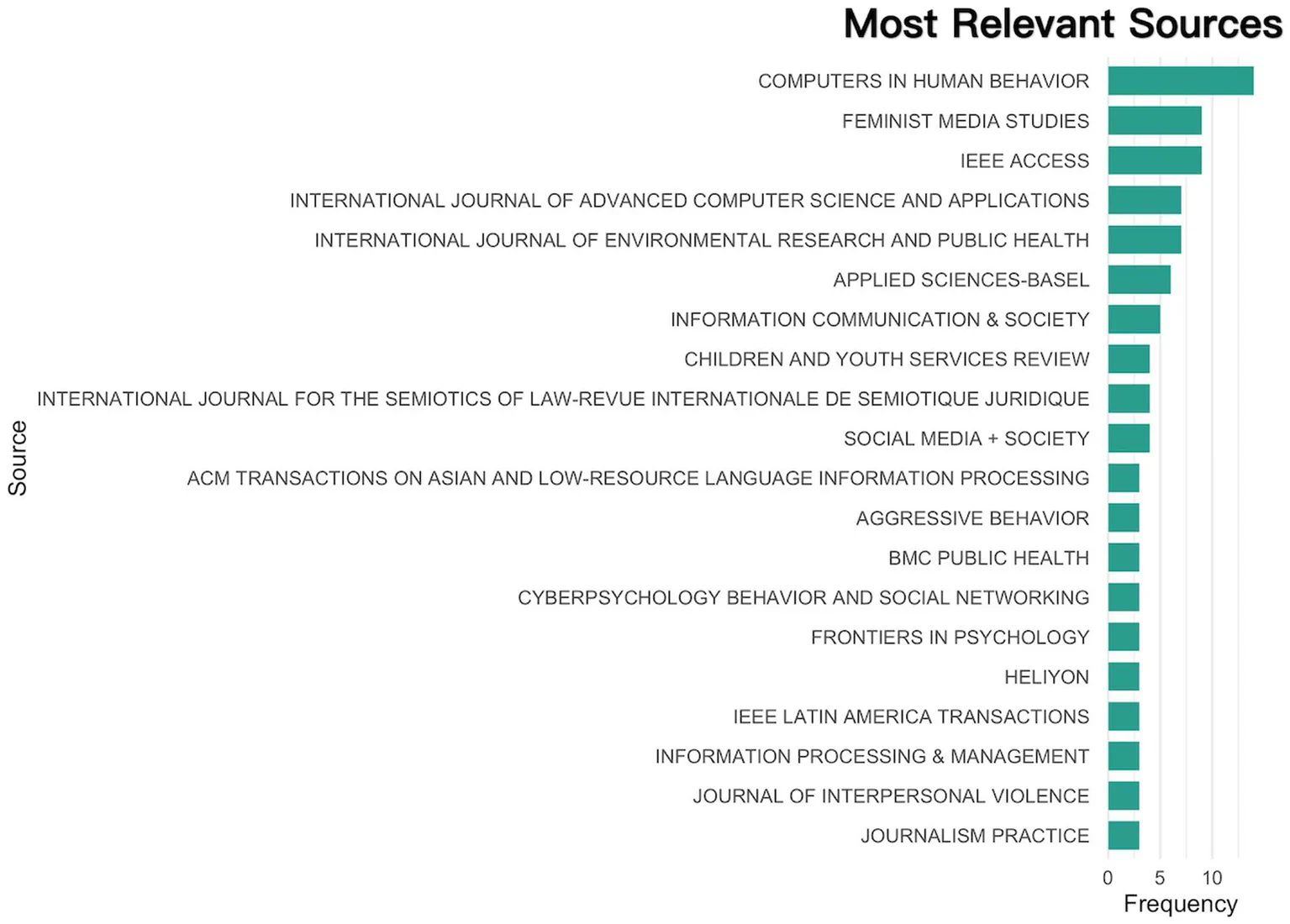
Most relevant publication sources in the 345-record corpus, ranked by number of documents. Bar length represents the number of corpus records published by each displayed source. This figure is a ranked performance summary rather than a network visualization; no co-occurrence threshold was applied.

A total of 1,015 authors contributed to the corpus. The most productive authors—Paulino, P. and Simão, A. M. V.—published five documents each, and Oliveira, S. published four, suggesting a distributed field of many small teams. Affiliation occurrences were led by the United States (128), Spain (62), India (55), Malaysia (38), Saudi Arabia (36), Portugal (35), China (34), Australia (30), South Korea (29), and the United Kingdom (29). Because full counting was used, totals exceed the number of publications. International co-authorship reached 62.03% (Table 2).

**Table 2:** Leading sources, authors, and countries in the corpus.

Dimension	Leading entities (documents or affiliation occurrences)
Sources	Computers in Human Behavior (14); Feminist Media Studies (9); IEEE Access (9); International Journal of Advanced Computer Science and Applications (7); International Journal of Environmental Research and Public Health (7)
Authors	Paulino, P. (5); Simão, A. M. V. (5); Oliveira, S. (4); several authors with three documents
Countries	USA (128); Spain (62); India (55); Malaysia (38); Saudi Arabia (36); Portugal (35); China (34); Australia (30); South Korea (29); United Kingdom (29)

#### 3.1.3 Country collaboration

The country collaboration network was anchored by the United States, which linked directly to partners across most regional communities (Figure 4). The United Kingdom anchored a Western European and Anglophone-adjacent community; Spain anchored an Iberian, Latin American, and Northern European community; India connected with Malaysia, Kazakhstan, Bangladesh, Yemen, Canada, and Australia; and Saudi Arabia connected with Pakistan, South Korea, Mexico, Jordan, Bahrain, Palestine, Tunisia, and Nigeria. China formed a more self-contained community linked mainly with Germany, the Czech Republic, and Bulgaria. The network therefore shows internationalization without uniform integration: multiple regional communities participate, but cross-community connectivity depends heavily on a small number of hubs.

**Figure 4:**
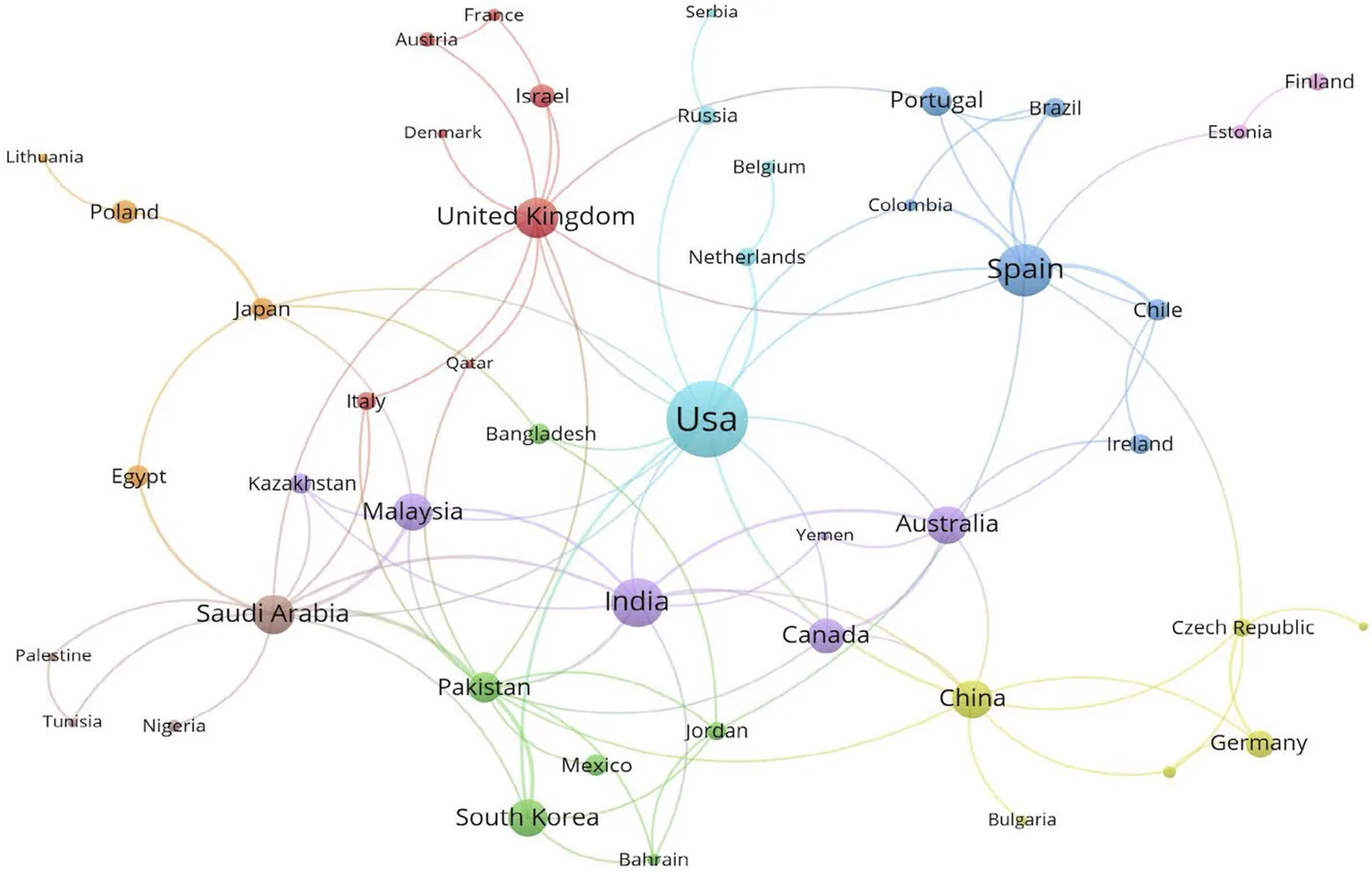
Country collaboration network for the 345-record corpus. Node size represents publication output associated with each country, node color indicates network-cluster membership, and links represent international co-authorship connections; thicker links indicate stronger collaboration. Countries with at least one publication were retained. Country counts use full counting, so a multinational paper contributes once to each represented country.

#### 3.1.4 Co-citation structure and foundational theories

The reference co-citation network resolved into two large, densely connected knowledge communities and a narrower bridge (Figure 5). One community was organized around psychosocial measurement, victimization, moral disengagement, developmental risk, and mental-health consequences. The second was organized around text classification, feature engineering, and automated detection. A smaller set of communication, discourse, gender, and platform studies linked the two. Within the retrieved corpus, this architecture indicates that work explaining cyberbullying processes and harms is related to, but not fully integrated with, work predicting whether a text belongs to an abusive class.

**Figure 5:**
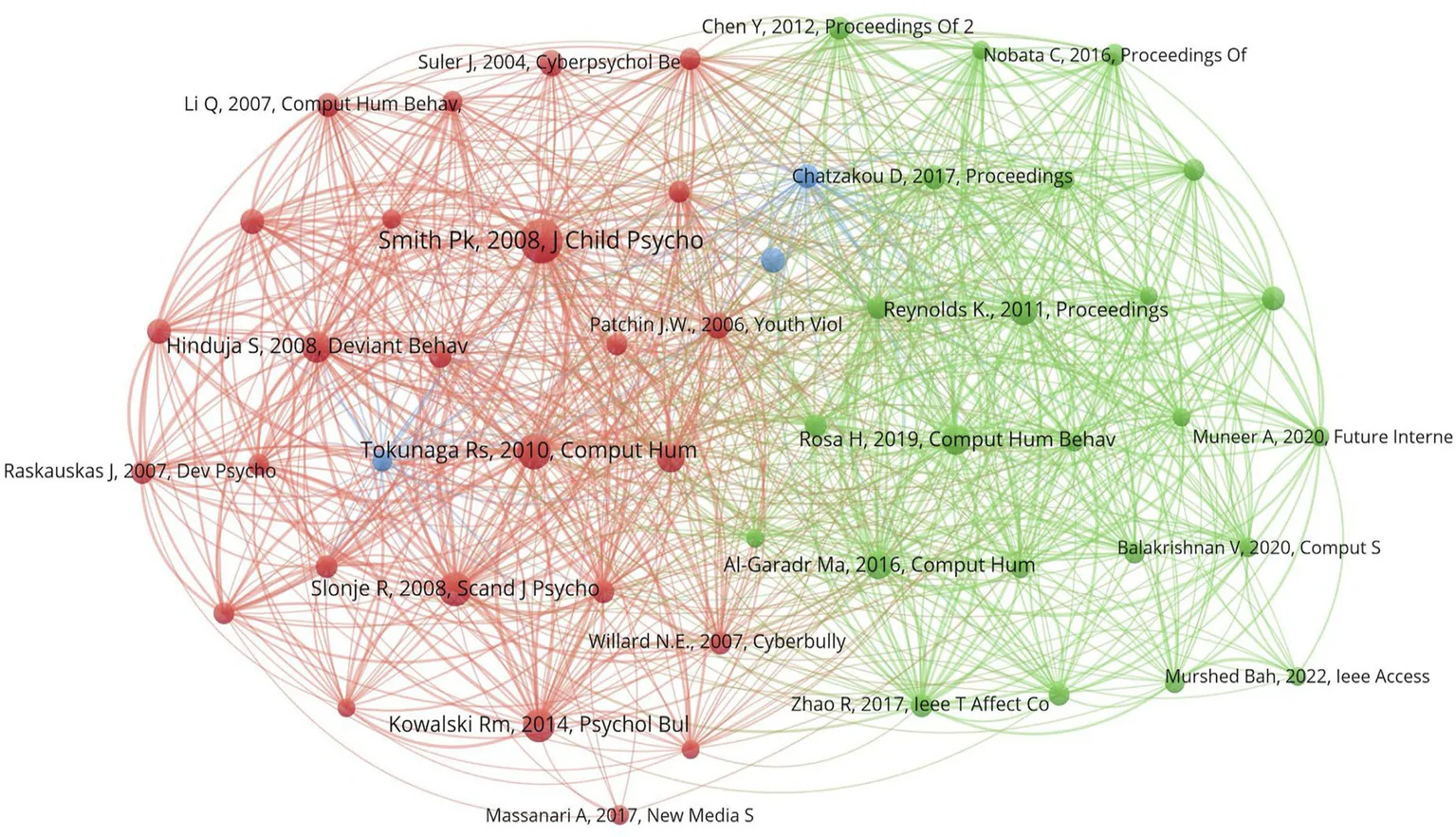
Reference co-citation network for the 345-record corpus. Node size represents cited-reference prominence, node color indicates cluster membership, and links represent co-citation relationships; thicker links indicate stronger co-citation connections. Cited references occurring at least 10 times were retained for display. CiteSpace used one-year time slices with g-index selection (k = 25), Pathfinder, and pruning of merged networks.

Two visible co-citation nodes are especially important for the present review. Suler (2004) supplies a media-psychological account of online disinhibition, while Kowalski et al. (2014) synthesize risk factors and psychological outcomes across the cyberbullying literature. Their structural prominence shows that foundational psychological explanations are visible at the cited-reference level within the retrieved corpus. However, their concepts are not consistently carried into lexical feature design, pragmatic annotation, or model evaluation. The network therefore reveals not an absence of psychological knowledge, but incomplete translation of that knowledge across disciplinary communities.

#### 3.1.5 Sensitivity analysis of corpus definition

A sensitivity analysis compared the primary broad corpus (*n* = 345) with a narrower cyberbullying-focused corpus containing 254 records (73.62% retained; 91 records excluded). Both corpora were analyzed using the same CiteSpace settings. The primary corpus network contained 357 nodes and 747 links (density = 0.0118), with modularity Q = 0.7819, weighted mean silhouette = 0.9236, and harmonic mean = 0.8468. The narrower network contained 332 nodes and 827 links (density = 0.0151), with Q = 0.7546, weighted mean silhouette = 0.9106, and harmonic mean = 0.8253. Both networks therefore showed clear modular differentiation and high within-cluster consistency. The larger number of links in the narrower network despite fewer nodes is compatible with the analysis procedure because CiteSpace reconstructs each corpus independently: g-index selection is applied separately within each one-year time slice before the slice-level networks are merged and pruned. A smaller but more topically concentrated corpus can therefore retain a denser set of recurrent co-citation relationships, so final link counts need not decrease proportionally with corpus size.

The author-keyword co-occurrence analysis was also repeated for the 254-record subcorpus using the same minimum-occurrence threshold of 10 as the primary corpus. The narrower network retained the principal structural contrast observed in the full corpus: psychosocial terms such as victimization, adolescents, school, prevalence, and behavior remained strongly interconnected, while computational terms including machine learning, deep learning, natural language processing, sentiment analysis, cyberbullying detection, and hate speech formed a distinct but connected area. Cyberbullying and social media occupied bridging positions between these strands. No explicitly pragmatic construct, such as implicature, stance, speech act, politeness, or impoliteness, reached the ≥10 display threshold in the narrower corpus. This result supports the interpretation that the limited high-frequency visibility of explicitly pragmatic terminology is not solely an artifact of the broader corpus definition (Supplementary Figure S3). Keyword burst analysis of the narrower corpus produced a closely related temporal pattern. Recent bursts remained concentrated in computational and outcome-oriented terminology including deep learning (strength = 4.22; 2021–2025) social media (2.48; 2021–2025) mental health (3.15; 2022–2025) cyberbullying detection (3.23; 2023–2025) and hate speech (2.86; 2023–2025). None of the 25 strongest bursts in the 254-record corpus was labeled with an explicitly pragmatic construct. Together with the co-citation and keyword-network comparisons this reproduces the principal asymmetry observed in the primary corpus: recent visibility was stronger for computational detection and psychosocial outcomes than for explicitly pragmatic terminology while individual terms and cluster prominence varied with corpus definition (Supplementary Figure S4).

Across the three sensitivity checks—co-citation clustering, author-keyword co-occurrence, and keyword bursts—the major computational/detection and psychosocial/behavioral strands remained visible, while explicitly pragmatic terminology remained comparatively weak at the selected high-frequency thresholds. The sensitivity analysis therefore supports robustness of the principal bibliometric pattern rather than exact replication of every node, label, or burst across the two corpus definitions.

#### 3.1.6 Journal citation pathways and keyword communities

The journal dual-map overlay (Figure 6) showed one dominant pathway from citing *Psychology*, *Education*, and *Health* journals to cited *Psychology*, *Education*, and *Social* journals (z ≈ 6.40; f ≈ 6,660). A secondary, thinner pathway connected Mathematics and Systems journals to Systems and Computing journals. No pathway of comparable weight linked the psychological and computational bases. Citation traffic was therefore more compartmentalized than the shared topic vocabulary might suggest.

**Figure 6:**
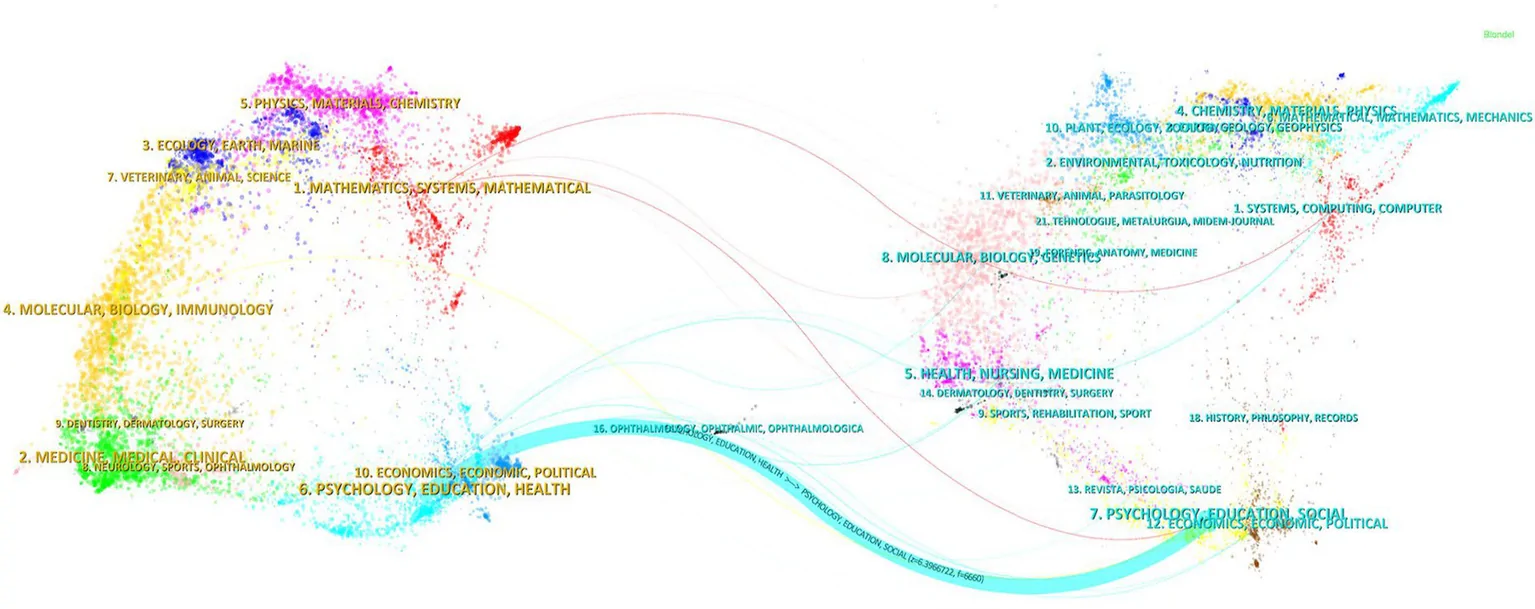
Journal dual-map overlay for the 345-record corpus. The left side represents the disciplinary domains of citing journals and the right side the domains of cited journals. Curved citation paths connect citing to cited domains, with more prominent paths representing stronger cross-domain citation trajectories. The overlay summarizes journal-level citation flows for the full corpus; no additional display threshold was specified for this visualization.

The author-keyword network divided into five high-frequency communities under the 10-occurrence display threshold (Figure 7). The largest psychosocial community contained victimization, adolescents, mental health, aggression, school, and gender. Computational and lexical communities contained hate speech, offensive language, sentiment analysis, machine learning, deep learning, natural language processing, feature extraction, and classification. Additional communities represented platform, identity, and critical-media concerns. Pragmatic constructs did not form an independent high-frequency community under this threshold; this limited network visibility should not be interpreted as evidence that pragmatic research is absent from the retrieved literature.

**Figure 7:**
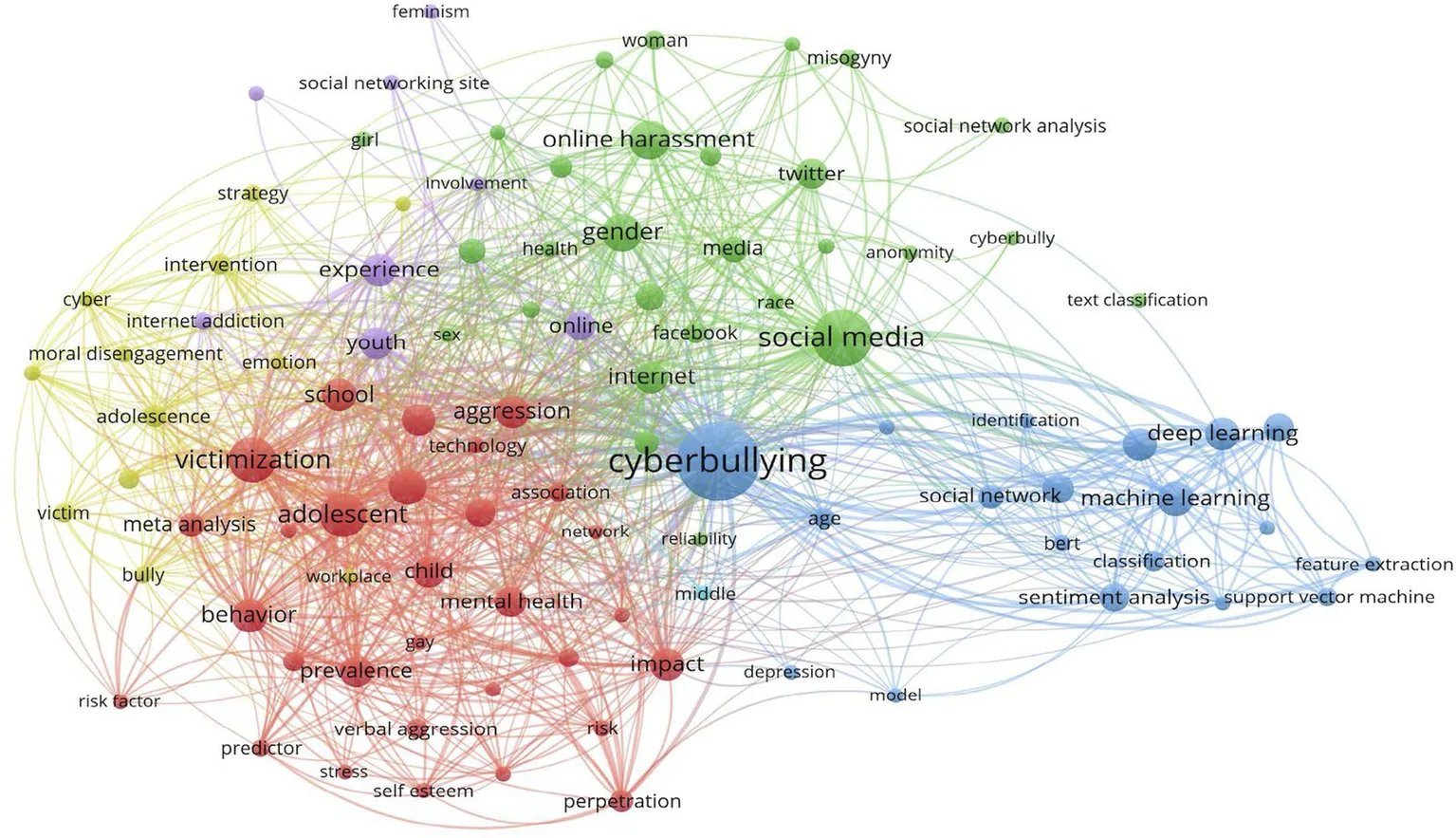
Author-keyword co-occurrence network for the primary 345-record corpus. Node size represents keyword occurrence frequency, node color indicates cluster membership, and links represent keyword co-occurrence relationships; thicker links indicate stronger co-occurrence. Only author keywords occurring at least 10 times were retained for visualization. The network was constructed in VOSviewer using association-strength normalization.

To distinguish sub-threshold visibility from substantive absence, author-keyword frequencies were inspected directly in the 345-record corpus. Sarcasm-related author keywords occurred five times in total (sarcasm detection, *n* = 4; multimodal sarcasm detection, *n* = 1), while impoliteness-related terms occurred three times (impoliteness, *n* = 2; mock impoliteness, *n* = 1). Irony-related detection terms occurred twice (irony detection, *n* = 1; irony-aware cyberbullying detection, *n* = 1). Stance (*n* = 1), speech act (*n* = 1), metapragmatics (*n* = 1), and politeness (*n* = 1) each appeared once. Implicature, presupposition, and audience uptake did not occur as author keywords. These counts refer specifically to author-keyword occurrences and therefore indicate indexing visibility rather than the substantive presence or absence of these concepts in article content.

To assess whether the low author-keyword frequencies underestimated the broader metadata visibility of pragmatic concepts, the same terms were counted at record level across titles, abstracts, author keywords, and Keywords Plus in the 345-record corpus (Supplementary Table S2). Pragmatic terminology was more visible in titles and abstracts than in indexing fields for several concepts. Stance occurred in 2 titles and 10 abstracts but in only 1 author-keyword record; sarcasm-related forms occurred in 5 titles and 14 abstracts compared with 5 author-keyword records; and irony-related forms appeared in 2 titles, 8 abstracts, 3 author-keyword records, and 1 Keywords Plus record. Pragmat* occurred in 2 titles and 9 abstracts but did not appear in DE or ID, while impoliteness appeared in 1 title, 3 abstracts, 3 author-keyword records, and 2 Keywords Plus records. By contrast, implicature-related forms did not occur in any of the four fields. These cross-field counts show that author-keyword frequencies alone understate the presence of some pragmatic concepts in record content, while explicitly pragmatic terminology remains comparatively limited across the corpus.

### 3.2 Temporal movement and emerging priorities

Temporally, the early period, roughly 2010 to 2015, was anchored in measurement, adolescent aggression, moral disengagement, and the first computational systems. Between 2016 and 2020, the retrieved literature diversified toward networked harassment, misogyny, platform culture, political hostility, and discourse communities. From 2021 onward, deep learning, feature extraction, BERT, explainable AI, multilingual detection, and mental health became increasingly visible.

The keyword time-overlay (Figure 8) makes this movement visible immediately. Psychosocial and developmental terms—adolescence, victim, bully, prevalence, victimization, verbal aggression, self-esteem, behavior, child—sit at the earlier end of the timeline. Gender, online harassment, discourse, and technology occupy the middle. Hate speech, machine learning, natural language processing, feature extraction, classification, and BERT dominate the later years.

**Figure 8:**
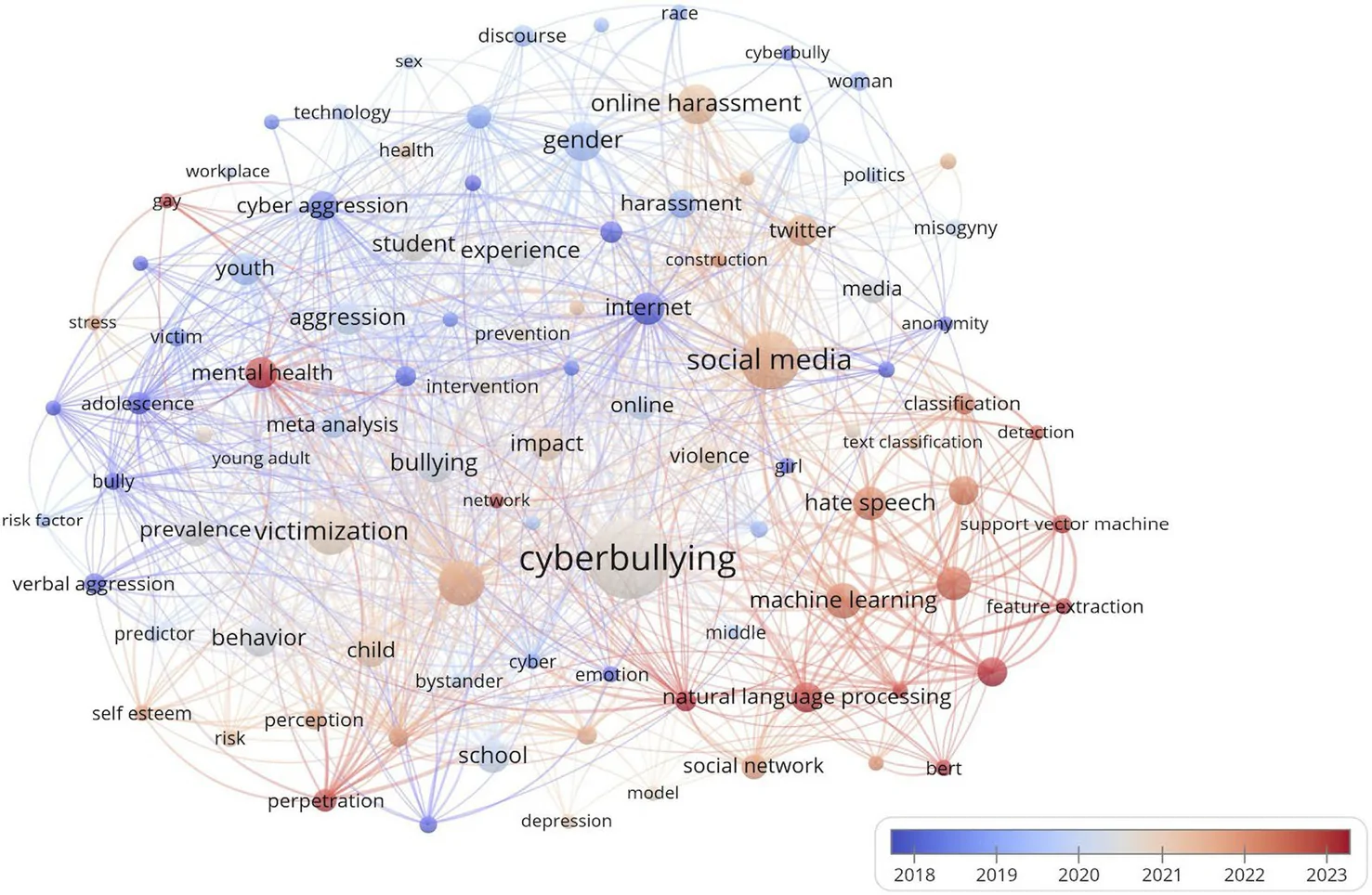
Author-keyword temporal overlay for the 345-record corpus. Node size represents keyword occurrence frequency, node color represents the average publication year of records containing the keyword, and links represent keyword co-occurrence relationships; thicker links indicate stronger co-occurrence. Author keywords occurring at least five times were retained to preserve lower-frequency temporal signals.

Burst analysis (Figure 9) sharpens this temporal pattern. Discourse burst only in 2016–2017, and gender and sex both ended their bursts before 2021. Deep learning, by contrast, remained in burst through the end of the study window (2022–2025, strength 4.51—the strongest in the table), as did hate speech (2023–2025, 3.39), mental health (2022–2025, 3.35), and cyberbullying detection (2023–2025). None of the 25 strongest bursts was labeled with an explicitly pragmatic construct. This pattern indicates that, within this search-defined corpus and under the reported burst settings, computational and outcome-oriented terminology gained recent visibility more rapidly than explicitly pragmatic terminology.

**Figure 9:**
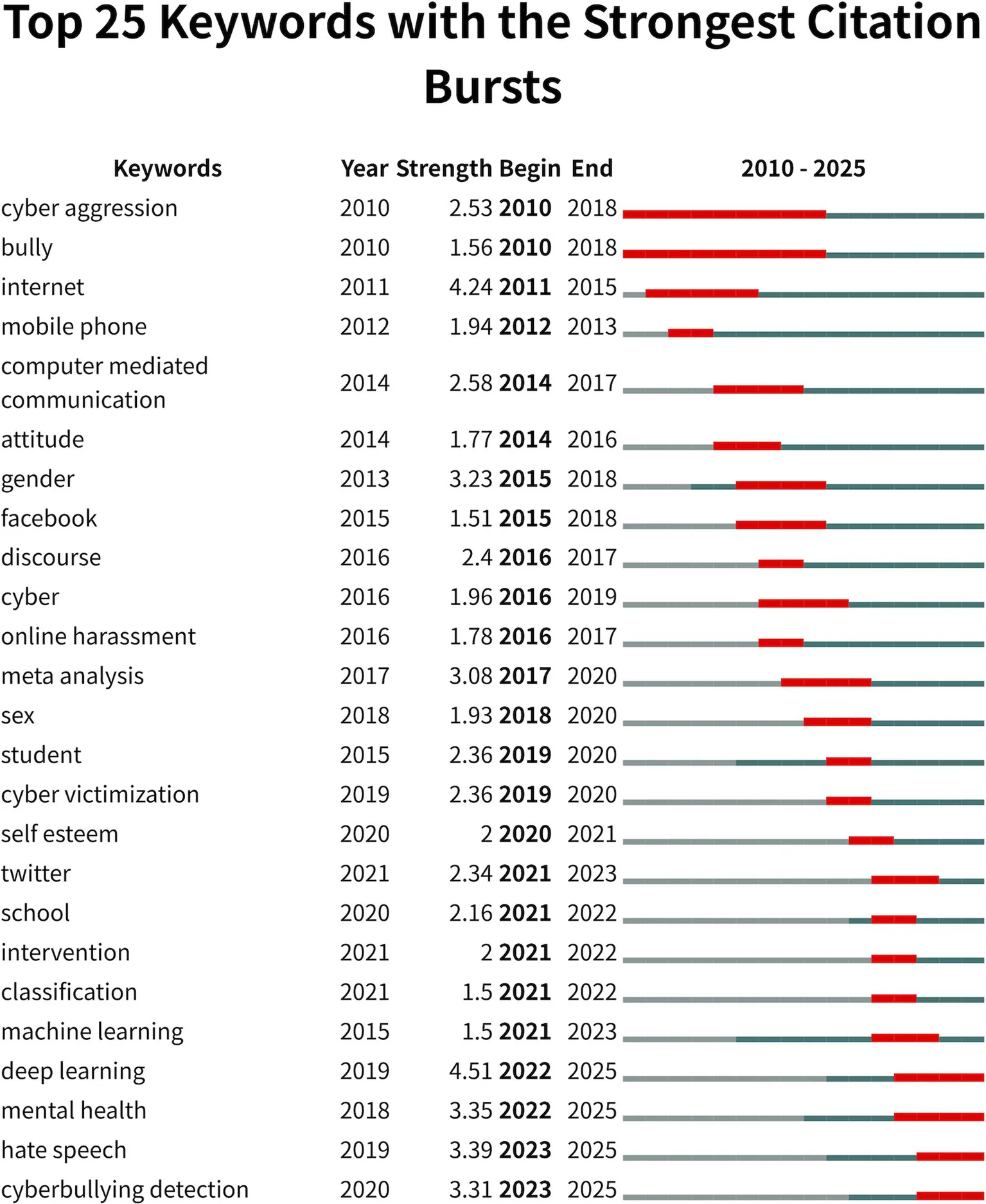
Keywords with the strongest citation bursts in the 345-record corpus across 2010–2025. Each row represents a keyword; burst strength quantifies the intensity of its temporary increase in prominence, and the highlighted interval marks the years during which the burst was active. The 25 strongest bursts identified by CiteSpace were retained for presentation.

## 4 Discussion

### 4.1 Principal findings and contribution to media psychology

The review identifies a rapidly expanding and internationally distributed field whose intellectual structure remains divided between psychosocial explanation and computational detection. Lexical-semantic research provides a partial bridge because lexical forms can function both as indicators of psychosocial harm and as model features. Pragmatic and discourse research is less strongly integrated into this bridge: it does not form a stable high-frequency keyword community, appears only intermittently in burst histories, and is rarely incorporated into large-scale annotation schemes. The principal contribution of the present review is therefore not simply to establish that cyberbullying is enacted through language, but to show that the mapped knowledge structure only partially connects media effects and psychological outcomes with the interpretive mechanisms through which linguistic forms acquire harmful meaning.

This separation is theoretically important because media effects are conditional rather than uniform. The Differential Susceptibility to Media Effects Model predicts variation according to dispositional, developmental, and social susceptibility, as well as cognitive, emotional, and excitative response states (Valkenburg and Peter, 2013). The same slur, the same sarcastic question, the same repeated label may therefore be appraised differently depending on prior victimization, the centrality of the targeted identity, relationship strength, platform norms, and audience response. Evidence that tie strength moderates the association between social-media use and cyberbullying is consistent with this perspective (Ni et al., 2025). Bibliometric fragmentation may therefore correspond to construct fragmentation when outcomes, message features, and interpretive processes are operationalized in separate research traditions.

### 4.2 Theory-informed interpretation through four analytical lenses

The following section provides theory-informed interpretation rather than additional bibliometric results. The keyword co-occurrence and co-citation structures revealed several interconnected research areas. To relate these mapped patterns to media psychology, the findings are interpreted through four non-mutually-exclusive analytical lenses: psychosocial victimization, lexical-semantic harm, pragmatics and discourse, and computational detection (Table 3). Three lenses corresponded closely to visible keyword communities, whereas pragmatic and discourse constructs were present but less consolidated in the high-frequency structures. External literature is used here for interpretive synthesis and is not treated as part of the bibliometric evidence.

**Table 3:** Theory-informed analytical lenses for interpreting the bibliometric structures.

Analytical lens	Indicative concepts	Interpretive emphasis	Media-psychological question
Psychosocial victimization	Victimization; adolescents; mental health; aggression; school; gender	Roles, prevalence, harm, developmental context, and well-being	Which cognitive, affective, relational, and social factors shape perpetration, victimization, and recovery?
Lexical-semantic harm	Hate speech; offensive language; slurs; sentiment; metaphor; embeddings	How harmful evaluation and group targeting are encoded	Which linguistic cues activate hostile intent, threat, stereotyping, or identity-based appraisal?
Pragmatics and discourse	Context; irony; sarcasm; humor; stance; implicature; interaction	Intent, uptake, sequence, community norms, and platformed meaning	How do users infer harmful force when meaning is indirect, quoted, cumulative, or contested?
Computational detection	Machine learning; deep learning; NLP; BERT; feature extraction; classification	Scalable identification and prediction performance	Do model labels and errors correspond to psychologically valid experiences of bullying and harm?

The four lenses are author-proposed, non-mutually-exclusive interpretive categories anchored to bibliometric structures and purposively selected illustrative records. They were not derived from formal full-text thematic coding or independent study-level coding.

#### 4.2.1 Psychosocial victimization

This is the broadest and most established of the four analytical lenses, by some distance. Measurement studies going back to Menesini et al. (2011) distinguished perpetrator, victim, and bystander roles, and a recent meta-analysis of bystander-intervention effectiveness suggests this three-role structure still holds up in current program design (Chen et al., 2025). Moral-disengagement research has examined how harmful conduct gets cognitively normalized (Lo Cricchio et al., 2021; Zhao and Yu, 2021), and meta-analytic work has connected cyberbullying to a range of individual, relational, and contextual correlates and outcomes (Kowalski et al., 2014). This is the broadest and most established of the four analytical lenses. Measurement studies dating to Menesini et al. (2011) distinguished perpetrator, victim, and bystander roles, and a recent meta-analysis of bystander-intervention effectiveness indicates that this three-role structure remains relevant to current program design (Chen et al., 2025). Moral-disengagement research has examined how harmful conduct becomes cognitively normalized (Lo Cricchio et al., 2021; Zhao and Yu, 2021), and meta-analytic work has linked cyberbullying to a range of individual, relational, and contextual correlates and outcomes (Kowalski et al., 2014). Recent evidence from a Brazilian public-school sample extends this outcome literature beyond predominantly Global North settings: Teixeira et al. (2025) examined 268 adolescents and found that cyberbullying victimization was associated with depressive symptoms, with the pattern differing by gender and symptom domain. Within this literature, language tends to appear as a behavioral indicator or survey category rather than as a stimulus whose form and inferred meaning may shape appraisal. Accordingly, this analytical lens provides substantial evidence on outcomes and psychological correlates but gives less attention to the linguistic properties of the harmful exposure itself.

#### 4.2.2 Lexical-semantic representation of harm

This analytical lens occupies an intermediate position between critical media research and computational detection and provides one of the clearest points of contact between these literatures. Critical and feminist scholarship has documented sexualized insults, racialized epithets, misogynistic labels, evaluative collocations, and the institutional responses or non-responses that follow harassment (Marwick and Caplan, 2018; Thompson, 2018; Lawson, 2018; Antunovic, 2019; Wright et al., 2020; Holton et al., 2023). More recent computational work adds an important qualification: harmful effects and classification errors are not driven only by explicit epithets, because racialized identity markers themselves can contribute to disproportionate misclassification of targeted speech as toxic (Harris et al., 2022). Computational studies operationalize related regularities through n-grams, sentiment scores, character sequences, embeddings, or attention-weighted features. This convergence makes lexical-semantic representation a productive bridge between socially situated accounts of harmful language and automated detection, although lexical features alone cannot establish pragmatic force or relational meaning.

#### 4.2.3 Pragmatics, discourse, and context

Pragmatic and discourse-oriented research challenges the assumption that harmful meaning can be inferred from an isolated sentence alone. Trolling, roast humor, sarcasm, coded misogyny, dog-whistles, counterspeech, and coordinated pile-ons require inference about intent, stance, participant rights and relationships, and local norms (Dynel and Poppi, 2019; Marwick and Caplan, 2018; Zhong et al., 2022). Recent cyberpragmatic research similarly emphasizes the importance of platform-specific context (Rhee, 2023). The relevant unit of analysis may therefore be a reply sequence, a thread, an ongoing relationship between accounts, or a networked audience rather than a single post. This analytical lens helps distinguish attack from banter, criticism from harassment, quotation from endorsement, and isolated hostility from cumulative interactional harm. Its comparatively limited visibility in high-frequency bibliometric structures should consequently be interpreted as limited indexing prominence rather than as evidence that contextual interpretation is unimportant.

#### 4.2.4 Computational detection

Within the retrieved corpus, computational detection became markedly more visible after 2020. The methods developed from commonsense reasoning and hand-engineered features to convolutional and recurrent networks, transformer-based embeddings, and ensembles (Salawu et al., 2020; Lu et al., 2020; Dewani et al., 2021; Chia et al., 2021; Iwendi et al., 2023; Mazari et al., 2024), while language coverage also broadened to Roman Urdu, Arabic, and other lower-resource settings. However, datasets, label definitions, and evaluation metrics remain insufficiently standardized for direct comparison across studies. High within-dataset accuracy may reflect stable lexical shortcuts rather than sensitivity to target, power, repetition, or intent. These errors are also unevenly distributed: research on abusive-language typologies and identity-term bias has shown that models can misclassify minority language, identity references, quotation, reclaimed language, and counterspeech. Computational progress is therefore substantial, but construct validity remains dependent on how cyberbullying is operationalized in training data and evaluation.

### 4.3 Integration of language and psychological process

Taken together, the four analytical lenses reveal a consistent pattern. Psychological processes are most explicit in survey and outcome research and least explicit in lexical feature engineering. Psychosocial studies measure moral disengagement, hostile attribution, self-esteem, distress, and mental-health outcomes, but they rarely identify which linguistic or pragmatic properties of an exposure contributed to the response. Lexical and computational studies, by contrast, describe message features in considerable detail while often leaving psychological harm implicit within categorical labels. Pragmatic research provides many of the interpretive relations needed to connect these strands, including speaker commitment, target, stance, sequence, audience, and uptake. Nevertheless, explicitly pragmatic constructs remained comparatively peripheral within the displayed high-frequency bibliometric structures.

This pattern does not indicate a shortage of psychological research; rather, it indicates limited integration across research traditions. Suler (2004) and Kowalski et al. (2014) are prominent, well-cited nodes in the co-citation structure, and moral disengagement is well represented. The less developed component is the translation of media-psychological constructs into annotation variables, contextual features, error categories, or hypotheses that can be tested across platforms. This integration gap motivates the conceptual framework developed in the Discussion.

### 4.4 Proposed conceptual language-to-harm framework

Drawing on three distinct evidential layers—the observed bibliometric structures, the theory-informed interpretation of representative records, and established media-psychological theories—we propose, rather than validate, a six-component conceptual language-to-harm framework (Figure 10). The bibliometric analyses directly establish only the mapped patterns of co-occurrence, co-citation, temporal prominence, and cross-domain visibility reported in the Results. They do not estimate directional or causal relationships among platform affordances, linguistic cues, pragmatic interpretation, psychological appraisal, behavioral and social responses, and cumulative harm. The arrows in Figure 10 therefore represent hypothesized conceptual pathways derived from the synthesis of the mapped literature and prior theory. They are intended to organize testable propositions for future empirical research rather than to function as an empirically validated path model. The feedback pathway involving repetition, audience uptake, and algorithmic amplification is likewise proposed as a hypothesis requiring direct longitudinal or interactional testing.

**Figure 10:**
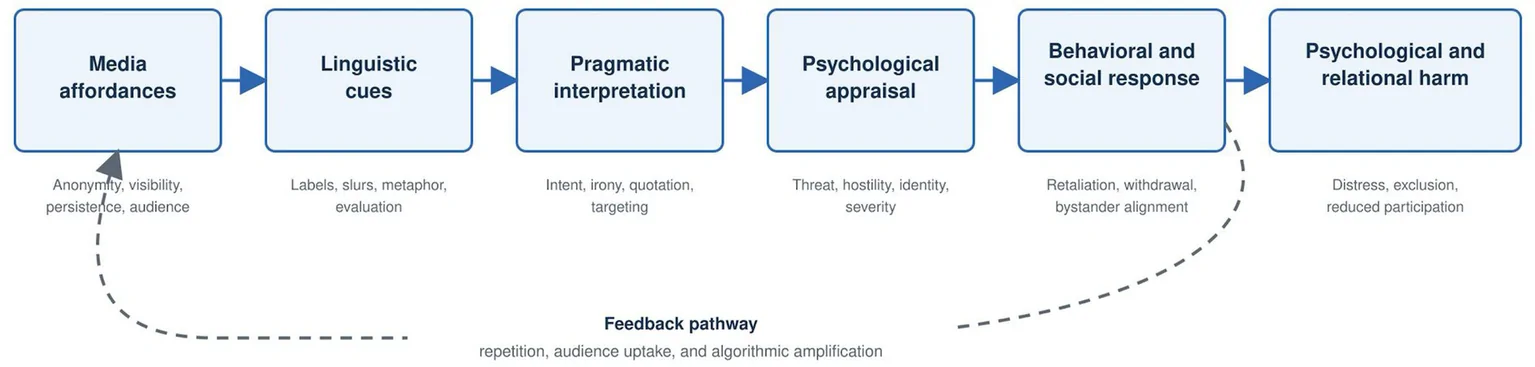
Proposed conceptual language-to-harm framework for media-psychological cyberbullying research. Solid arrows denote the proposed directional relationships; the dashed feedback pathway denotes the proposed role of repetition, audience uptake, and algorithmic amplification. See Section 4.4 for the framework’s evidential status and interpretation.

The framework comprises six linked components. At the first component, platform affordances may condition behavior and exposure: anonymity, invisibility, asynchronicity, persistence, searchability, and scalable audiences may reduce restraint or alter the immediacy of social feedback (Suler, 2004). At the second component, lexical and grammatical choices provide potential linguistic cues—for example, labels may categorize a target, metaphors may dehumanize, intensifiers may strengthen evaluation, and creative spellings may evade moderation filters. At the third component, these cues require pragmatic interpretation to be understood as, for example, a committed attack, irony, quotation, banter, threat, or counterspeech. Walther’s (1996) account of computer-mediated communication supports this interpretive emphasis by showing that users adapt to the verbal and relational cues available online rather than simply losing social information.

Within the proposed framework, the fourth component is psychological appraisal. The General Aggression Model provides a theoretical basis for hypothesizing that situational inputs and person factors may operate through cognition, affect, arousal, and appraisal before behavioral responses occur (Anderson and Bushman, 2002). For perpetrators, hostile scripts and anger may increase the likelihood of aggressive responding; for targets, perceived hostility, identity threat, controllability, and public exposure may contribute to distress. Moral disengagement provides a complementary theoretical account of how self-sanctions may be weakened through sanitized labeling, diffusion of responsibility, and victim blaming (Bandura, 1999). The fifth and sixth components represent possible behavioral and social responses and the accumulation of psychological or relational harm over time. Retaliation, withdrawal, reporting, defending, reinforcement, or non-intervention is therefore treated as theoretically plausible response pathways rather than as effects estimated by the present bibliometric analysis. Existing experimental and survey research on bystanders supports the relevance of social norms, felt responsibility, and perceived severity to intervention decisions (Bastiaensens et al., 2014; Van Cleemput et al., 2014), but the full sequence proposed in Figure 10 remains to be tested directly.

### 4.5 Why lexical analysis requires pragmatics

Lexical evidence remains indispensable: many attacks use repeated evaluative forms, sexualized or racialized labels, profanity, dehumanizing metaphor, and identity terms. However, a token cannot establish illocutionary force by itself. Quotation can reproduce a slur specifically to condemn it. Reclaimed language can express solidarity, and sarcasm can flip literal polarity on its head. Conversely, an apparently ordinary label may become abusive through accumulated repetition, while coordinated exclusion, insinuation, or dog-whistles may cause harm without any taboo word being present (Hardaker, 2010; Marwick and Caplan, 2018; Dynel and Poppi, 2019; Zhong et al., 2022).

The burst results further illustrate this gap. Deep learning and detection remained prominent through 2025, whereas discourse did not reappear after its 2016–2017 burst, and none of the strongest bursts directly identified stance, implicature, speech act, or audience uptake. Increasing model complexity without enriching contextual representation may therefore treat pragmatic ambiguity as residual noise rather than as analytically meaningful variation. Future corpora should incorporate thread structure, reply to relations, timestamps, speaker-target history, platform visibility, and audience response, subject to appropriate privacy safeguards. Annotation schemes should likewise distinguish target type, speaker commitment, quotation, irony, humor, perceived severity, and relationship history. Such design choices would make psychologically meaningful model errors available for direct inspection rather than obscuring them within aggregate accuracy measures.

### 4.6 Collective amplification, bystanders, and relational harm

A defining distinction between cyberbullying and a single offensive comment is that harm may be relational, repetitive, and collectively amplified. Moral disengagement is not limited to intrapersonal cognition; diffusion of responsibility can allow individual participants to treat small contributions as inconsequential even when their cumulative effect becomes severe (Bandura, 1999; Zhao and Yu, 2021). Likes, reposts, tags, and replies can function as forms of behavioral alignment by increasing visibility and signaling normative approval. The psychological meaning of a hostile message may therefore depend partly on patterns of participation, non-intervention, and repeated platform exposure.

Bystander research provides a direct conceptual bridge between pragmatic uptake and media effects because audiences interpret severity, responsibility, social norms, and relational cues before deciding whether to defend, reinforce, ignore, or report harmful content (Bastiaensens et al., 2014; Van Cleemput et al., 2014). This relationship remains weakly represented in the keyword and computational structures mapped here. Future research should therefore operationalize audience uptake both as a psychological process and as an observable interactional phenomenon.

### 4.7 Implications for detection, moderation, and well-being

Detection systems have clear practical value, but sentence-level accuracy alone is an insufficient evaluation criterion. False negatives can leave users exposed to coded or cumulative aggression that remains undetected, whereas false positives can suppress reclaimed identity language, minority vernaculars, quotation, humor, or activist counterspeech. Research has demonstrated unintended identity bias in toxicity classification and has argued for more nuanced error metrics (Borkan et al., 2019; Sap et al., 2019). Psychologically valid evaluation should therefore assess whether model decisions correspond to perceived intent, severity, target vulnerability, repetition, and likely harm across different users and communities rather than only in aggregate.

Human–AI moderation may be more effectively designed as an interpretive system rather than as a profanity filter. Automated models can prioritize content and identify interactional escalation, while human reviewers assess context, relationships, and contested meaning. Moderation decisions should provide interpretable evidence for classification, and users should have mechanisms to contest misclassification. This is particularly important in multilingual settings, where politeness systems, code-switching, taboo formation, and identity labels cannot be reduced to a translated English lexicon. Such an approach may support mental health and participation by reducing both under-enforcement and discriminatory over-enforcement (Schoenebeck et al., 2023; Deng et al., 2026).

### 4.8 Research agenda

Based on the bibliometric findings and theory-informed interpretation presented above, Table 4 links each future-research priority explicitly to a corresponding evidential pattern identified in the present review. The recommendations are therefore not presented as generic prescriptions; rather, each addresses a specific limitation, fragmentation, or methodological imbalance observed within the retrieved corpus. These priorities are not direct outputs of the co-occurrence or co-citation analyses; instead, they translate the mapped patterns and interpretive gaps into targeted methodological recommendations.

**Table 4:** Research agenda grounded in findings from the present review.

Priority	Evidence/rationale from this review	Recommended design	Expected contribution
Context-rich longitudinal corpora	Pragmatic and interactional constructs showed limited prominence in the thresholded bibliometric structures, while computational research was dominated by text-classification approaches that often abstracted messages from relational and temporal context.	Retain threads, reply to relations, timestamps, visibility, repetition, and speaker–target history with privacy safeguards	Distinguish isolated offense from sustained relational aggression and cumulative exposure
Pragmatic and psychological annotation	The review identified limited integration between linguistic/pragmatic representations and psychological processes: psychosocial studies emphasized appraisal, victimization, moral disengagement, and harm, whereas lexical and computational studies more often operationalized message features without corresponding psychological variables.	Annotate stance, speech act, implicature, quotation, humor, target, perceived intent, severity, threat, and audience uptake	Connect linguistic evidence with appraisal, behavior, and harm
Multilingual and cross-platform validation	Corpus limitation and theory-informed interpretation, rather than a mapped bibliometric result. The present corpus was restricted to English-language records from a single database (Web of Science), while the interpreted literature indicates that linguistic meaning and harmfulness are conditioned by platform, relational, and sociocultural context.	Use language-specific expertise, code-switching protocols, measurement invariance, and platform comparison	Reduce Anglocentric bias and false equivalence across communities
Fairness-aware human–AI evaluation	Cited classifier-bias literature, rather than a mapped bibliometric result. Prior studies cited in the review document context loss, identity-term bias, and classification errors involving minority language, quotation, reclaimed language, and counterspeech, supporting the need for fairness-aware evaluation.	Report subgroup and identity-term errors; test context-sensitive appeals and human review	Protect targets without disproportionately silencing vernacular, counterspeech, or dissent
Reproducible bibliometrics	The sensitivity analysis showed broad structural robustness across corpus definitions, while individual cluster labels and topic visibility changed with corpus boundaries and threshold settings.	Archive exact queries, export dates, screening decisions, counting rules, thresholds, code, and representative documents	Enable replication, critical appraisal, and longitudinal updating

### 4.9 Limitations

Several limitations delimit the interpretation of the present findings. First, as specified in Section 2.2, the corpus is restricted to English-language records indexed in the Web of Science Core Collection. Relevant work indexed only in Scopus, discipline-specific databases, regional indexes, or non-English outlets may have been omitted, particularly in linguistics and pragmatics where publication venues are heterogeneous. Moreover, geographically diverse authorship in the country collaboration network should not be equated with multilingual or culturally representative evidence. Research conducted in underrepresented settings, including recent work with Brazilian public-school adolescents (Teixeira et al., 2025), illustrates the importance of extending cyberbullying research beyond predominantly Anglophone and Global North contexts.

Second, citation and network measures are affected by bibliographic age, metadata quality, normalization procedures, node-selection rules, and display thresholds. Older publications have had more time to accumulate citations, while low-frequency concepts may remain invisible in thresholded networks even when relevant studies are present in the corpus. Accordingly, the limited prominence of pragmatic constructs in the displayed maps should be interpreted as limited bibliometric visibility under the reported settings rather than evidence that pragmatic research is absent from the literature.

Third, the interpretive component relies on bibliometric structures and purposively selected representative records rather than exhaustive study-level content coding; the selection and assignment procedure is detailed in Section 2.4. The evidential status of the proposed language-to-harm framework is defined in Section 4.4.

Finally, the primary search strategy deliberately included adjacent online-aggression terminology, including online harassment, cyber aggression, and verbal abuse, in order to capture linguistically and computationally relevant scholarship surrounding cyberbullying. This breadth introduces construct heterogeneity. The sensitivity analysis partly addresses this concern: comparison of the primary 345-record corpus with the narrower 254-record cyberbullying-focused subcorpus showed broad structural robustness while also demonstrating that corpus definition can alter the visibility and labeling of individual clusters. The sensitivity analysis therefore strengthens confidence in the principal intellectual architecture but does not remove database-coverage, language, metadata, or threshold-related limitations.

## 5 Conclusion

Within the analyzed corpus, lexical and pragmatic cyberbullying research expanded substantially between 2010 and 2025, but its development was uneven across intellectual areas. The bibliometric results show strong and comparatively well-established psychosocial and computational strands, while explicitly pragmatic constructs remain less consolidated in the high-frequency mapped structures. Taken together with the theory-informed interpretation, these patterns identify a conceptual and bibliometric gap: linguistic and pragmatic representations are not yet systematically integrated with the psychological processes, outcomes, and platform conditions emphasized elsewhere in the corpus.

To address this gap, the review proposes a conceptual language-to-harm framework linking platform affordances, linguistic cues, pragmatic interpretation, psychological appraisal, behavioral and social responses, and cumulative psychological and relational harm. Future studies should test these proposed relationships using context-rich longitudinal and interactional data, pragmatic and psychological annotation, multilingual and cross-platform validation, and fairness-aware human-AI evaluation.

## Data Availability

The raw data supporting the conclusions of this article will be made available by the authors, without undue reservation.
