# Supplementary material for: Language and context in cyberbullying research: bibliometric mapping and conceptual integration of lexical, pragmatic, and media-psychological perspectives (2010–2025)
Source: Front Psychol. 2026 Oct 21;17:1963458. doi: 10.3389/fpsyg.2026.1963458 (PMC13731407; doi:10.3389/fpsyg.2026.1963458)
Supplement: Supplementary file 1 [file Table_1.DOCX]

**Supplementary Table S1. Records excluded from the narrow cyberbullying-focused sensitivity subcorpus (n = 91)**

Exclusion rule: records from the original 345-record Web of Science export were excluded from the narrow sensitivity subcorpus when the case-insensitive pattern \bcyber-?bully did not occur in any of the target fields TI (title), AB (abstract), DE (author keywords), or ID (Keywords Plus). Matches occurring only in cited references (CR) or other non-target fields were not counted. The rule intentionally did not capture the spaced form “cyber bullying”.

| **No.** | **WoS UT** | **Author(s)** | **Year** | **Title** | **Source** | **DOI** | **Exclusion reason** |
| --- | --- | --- | --- | --- | --- | --- | --- |
| 1 | WOS:000639170600044 | Crane, CA Wiernik, BM Berbary, CM Crawford, M Schlauch, RC Easton, CJ | 2021 | A meta-analytic review of the relationship between cyber aggression and substance use | DRUG AND ALCOHOL DEPENDENCE | 10.1016/j.drugalcdep.2021.108510 | No match for ‘cyberbully’ or ‘cyber-bully’ in TI/AB/DE/ID |
| 2 | WOS:001484200200001 | Chen, YL Wang, LB Guo, SY Gu, Q | 2025 | Violent or competitive? Unpacking adolescent cyber-aggressive behavior in text, video, and game context | FRONTIERS IN PSYCHOLOGY | 10.3389/fpsyg.2025.1577717 | No match for ‘cyberbully’ or ‘cyber-bully’ in TI/AB/DE/ID |
| 3 | WOS:001274730200001 | Valcarcel, AS Fernandez, EG Rivino, BRT | 2024 | Perceptions, experiences, and positions on gender violence in social media. Results of a survey of Andalusian young people | COMUNICACION Y SOCIEDAD-GUADALAJARA | 10.32870/cys.v2024.8676 | No match for ‘cyberbully’ or ‘cyber-bully’ in TI/AB/DE/ID |
| 4 | WOS:000274644800001 | Pornari, CD Wood, J | 2010 | Peer and Cyber Aggression in Secondary School Students: The Role of Moral Disengagement, Hostile Attribution Bias, and Outcome Expectancies | AGGRESSIVE BEHAVIOR | 10.1002/ab.20336 | No match for ‘cyberbully’ or ‘cyber-bully’ in TI/AB/DE/ID |
| 5 | WOS:000517424700008 | Wright, MF | 2020 | The Role of Technologies, Behaviors, Gender, and Gender Stereotype Traits in Adolescents' Cyber Aggression | JOURNAL OF INTERPERSONAL VIOLENCE | 10.1177/0886260517696858 | No match for ‘cyberbully’ or ‘cyber-bully’ in TI/AB/DE/ID |
| 6 | WOS:000541057400001 | Sainju, KD | 2020 | Beyond the Schoolyard: A Multilevel Examination of Individual, School and School District Variables Associated with Traditional and Cyber Peer Aggression | CHILD & YOUTH CARE FORUM | 10.1007/s10566-020-09555-4 | No match for ‘cyberbully’ or ‘cyber-bully’ in TI/AB/DE/ID |
| 7 | WOS:000399094000007 | Keum, BT Miller, MJ | 2017 | Racism in Digital Era: Development and Initial Validation of the Perceived Online Racism Scale (PORS v1.0) | JOURNAL OF COUNSELING PSYCHOLOGY | 10.1037/cou0000205 | No match for ‘cyberbully’ or ‘cyber-bully’ in TI/AB/DE/ID |
| 8 | WOS:000644284000001 | Sullivan, TN Farrell, AD Sutherland, KS Behrhorst, KL Garthe, RC Greene, A | 2021 | Evaluation of the Olweus Bullying Prevention Program in US Urban Middle Schools Using a Multiple Baseline Experimental Design | PREVENTION SCIENCE | 10.1007/s11121-021-01244-5 | No match for ‘cyberbully’ or ‘cyber-bully’ in TI/AB/DE/ID |
| 9 | WOS:000356786700010 | Chou, WYS Prestin, A Kunath, S | 2014 | Obesity in social media: a mixed methods analysis | TRANSLATIONAL BEHAVIORAL MEDICINE | 10.1007/s13142-014-0256-1 | No match for ‘cyberbully’ or ‘cyber-bully’ in TI/AB/DE/ID |
| 10 | WOS:000684444100001 | Rodis, PDCI | 2021 | Let's (re)tweet about racism and sexism: responses to cyber aggression toward Black and Asian women | INFORMATION COMMUNICATION & SOCIETY | 10.1080/1369118X.2021.1962948 | No match for ‘cyberbully’ or ‘cyber-bully’ in TI/AB/DE/ID |
| 11 | WOS:000417656700015 | Bogolyubova, O Panicheva, P Tikhonov, R Ivanov, V Ledovaya, Y | 2018 | Dark personalities on Facebook: Harmful online behaviors and language | COMPUTERS IN HUMAN BEHAVIOR | 10.1016/j.chb.2017.09.032 | No match for ‘cyberbully’ or ‘cyber-bully’ in TI/AB/DE/ID |
| 12 | WOS:000852127600001 | Mundra, S Mittal, N | 2023 | CMHE-AN: Code mixed hybrid embedding based attention network for aggression identification in hindi english code-mixed text | MULTIMEDIA TOOLS AND APPLICATIONS | 10.1007/s11042-022-13668-4 | No match for ‘cyberbully’ or ‘cyber-bully’ in TI/AB/DE/ID |
| 13 | WOS:000219134700005 | Leisring, PA Giumetti, GW | 2014 | Sticks and Stones May Break My Bones, But Abusive Text Messages Also Hurt: Development and Validation of the Cyber Psychological Abuse Scale | PARTNER ABUSE | 10.1891/1946-6560.5.3.323 | No match for ‘cyberbully’ or ‘cyber-bully’ in TI/AB/DE/ID |
| 14 | WOS:000971928500001 | Azeez, NA Fadhal, E | 2023 | Classification of Virtual Harassment on Social Networks Using Ensemble Learning Techniques | APPLIED SCIENCES-BASEL | 10.3390/app13074570 | No match for ‘cyberbully’ or ‘cyber-bully’ in TI/AB/DE/ID |
| 15 | WOS:000462222500003 | Wagner, A | 2019 | E-victimization and e-predation theory as the dominant aggressive communication: the case of cyber bullying | SOCIAL SEMIOTICS | 10.1080/10350330.2019.1587832 | No match for ‘cyberbully’ or ‘cyber-bully’ in TI/AB/DE/ID; target field contains spaced form ‘cyber bullying’, intentionally outside the narrow regex |
| 16 | WOS:000980825100002 | Gosse, C O'Meara, V Hodson, J Veletsianos, G | 2024 | Too rigid, too big, and too slow: institutional readiness to protect and support faculty from technology facilitated violence and abuse | HIGHER EDUCATION | 10.1007/s10734-023-01043-7 | No match for ‘cyberbully’ or ‘cyber-bully’ in TI/AB/DE/ID; target field contains spaced form ‘cyber bullying’, intentionally outside the narrow regex |
| 17 | WOS:000338432400020 | Doobo, S | 2014 | THE CYBER BULLYING OF POP STAR TABLO AND SOUTH KOREAN SOCIETY: HEGEMONIC DISCOURSES ON EDUCATIONAL BACKGROUND AND MILITARY SERVICE | ACTA KOREANA | 10.18399/acta.2014.17.1.018 | No match for ‘cyberbully’ or ‘cyber-bully’ in TI/AB/DE/ID; target field contains spaced form ‘cyber bullying’, intentionally outside the narrow regex |
| 18 | WOS:000348889700003 | Lee, S | 2015 | Analyzing negative SNS behaviors of elementary and middle school students in Korea | COMPUTERS IN HUMAN BEHAVIOR | 10.1016/j.chb.2014.10.014 | No match for ‘cyberbully’ or ‘cyber-bully’ in TI/AB/DE/ID; target field contains spaced form ‘cyber bullying’, intentionally outside the narrow regex |
| 19 | WOS:000418964200006 | Yam, KC | 2018 | The Effects of Thought Suppression on Ethical Decision Making: Mental Rebound Versus Ego Depletion | JOURNAL OF BUSINESS ETHICS | 10.1007/s10551-015-2944-2 | No match for ‘cyberbully’ or ‘cyber-bully’ in TI/AB/DE/ID; target field contains spaced form ‘cyber bullying’, intentionally outside the narrow regex |
| 20 | WOS:001512102800001 | Aziz, A | 2025 | I am the price of your freedom: Gender, Islam, and cyber-harassment in the aftermath of France's Affaire Mila | EUROPEAN JOURNAL OF WOMENS STUDIES | 10.1177/13505068251348320 | No match for ‘cyberbully’ or ‘cyber-bully’ in TI/AB/DE/ID |
| 21 | WOS:000390354000001 | Behm-Morawitz, E Schipper, S | 2016 | Sexing the Avatar Gender, Sexualization, and Cyber-Harassment in a Virtual World | JOURNAL OF MEDIA PSYCHOLOGY-THEORIES METHODS AND APPLICATIONS | 10.1027/1864-1105/a000152 | No match for ‘cyberbully’ or ‘cyber-bully’ in TI/AB/DE/ID |
| 22 | WOS:000443662300003 | Lee, HS Lee, HR Park, JU Han, YS | 2018 | An abusive text detection system based on enhanced abusive and non-abusive word lists | DECISION SUPPORT SYSTEMS | 10.1016/j.dss.2018.06.009 | No match for ‘cyberbully’ or ‘cyber-bully’ in TI/AB/DE/ID; target field contains spaced form ‘cyber bullying’, intentionally outside the narrow regex |
| 23 | WOS:001290238400001 | Iqbal, I Chinnasamy, S | 2024 | Navigating misogynistic cyber harassment: Impacts and challenges faced by Pakistani female journalists | SEARCH-JOURNAL OF MEDIA AND COMMUNICATION RESEARCH | 10.58946/search-16.2.P4 | No match for ‘cyberbully’ or ‘cyber-bully’ in TI/AB/DE/ID |
| 24 | WOS:001335820200060 | Gajalakshmi, G Meenakshi, S | 2023 | Understanding the psycho-social problems of vulnerable adolescent girls and effect of intervention through life skill training | JOURNAL OF EDUCATION AND HEALTH PROMOTION | 10.4103/jehp.jehp_612_23 | No match for ‘cyberbully’ or ‘cyber-bully’ in TI/AB/DE/ID; target field contains spaced form ‘cyber bullying’, intentionally outside the narrow regex |
| 25 | WOS:000212384300006 | Halder, D Jaishankar, K | 2011 | Cyber Gender Harassment and Secondary Victimization: A Comparative Analysis of the United States, the UK, and India | VICTIMS & OFFENDERS | 10.1080/15564886.2011.607402 | No match for ‘cyberbully’ or ‘cyber-bully’ in TI/AB/DE/ID |
| 26 | WOS:001148626000001 | Björkenfeldt, O Gustafsson, L | 2023 | Impoliteness and morality as instruments of destructive informal social control in online harassment targeting Swedish journalists | LANGUAGE & COMMUNICATION | 10.1016/j.langcom.2023.11.002 | No match for ‘cyberbully’ or ‘cyber-bully’ in TI/AB/DE/ID |
| 27 | WOS:000516827400200 | Yang, X Jiang, XW Mo, PKH Cai, Y Ma, L Lau, JTF | 2020 | Prevalence and Interpersonal Correlates of Internet Gaming Disorders among Chinese Adolescents | INTERNATIONAL JOURNAL OF ENVIRONMENTAL RESEARCH AND PUBLIC HEALTH | 10.3390/ijerph17020579 | No match for ‘cyberbully’ or ‘cyber-bully’ in TI/AB/DE/ID |
| 28 | WOS:000391110900036 | Surroop, K Canoo, K Pudaruth, S | 2016 | A Novel Position-based Sentiment Classification Algorithm for Facebook Comments | INTERNATIONAL JOURNAL OF ADVANCED COMPUTER SCIENCE AND APPLICATIONS |  | No match for ‘cyberbully’ or ‘cyber-bully’ in TI/AB/DE/ID; target field contains spaced form ‘cyber bullying’, intentionally outside the narrow regex |
| 29 | WOS:000992610000002 | Beltrán, SA Morales, SS Conde, MH | 2023 | [en] Interference in feminist participation and deliberation by digital facism | TEKNOKULTURA: REVISTA DE CULTURA DIGITAL Y MOVIMIENTOS SOCIALES | 10.5209/tekn.81002 | No match for ‘cyberbully’ or ‘cyber-bully’ in TI/AB/DE/ID |
| 30 | WOS:001136028300004 | Sampaio-Dias, S Silveirinha, MJ Garcez, B Subtil, F Miranda, J Cerqueira, C | 2024 | "Journalists are Prepared for Critical Situations … but We are Not Prepared for This": Empirical and Structural Dimensions of Gendered Online Harassment | JOURNALISM PRACTICE | 10.1080/17512786.2023.2250755 | No match for ‘cyberbully’ or ‘cyber-bully’ in TI/AB/DE/ID |
| 31 | WOS:000726985200003 | McCambridge, L | 2022 | Describing the voice of online bullying: An analysis of stance and voice type in YouTube comments | DISCOURSE CONTEXT & MEDIA | 10.1016/j.dcm.2021.100552 | No match for ‘cyberbully’ or ‘cyber-bully’ in TI/AB/DE/ID |
| 32 | WOS:000860520900003 | Demir, Y Ayhan, B | 2022 | Being a Female Sports Journalist on Twitter: Online Harassment, Sexualization, and Hegemony | INTERNATIONAL JOURNAL OF SPORT COMMUNICATION | 10.1123/ijsc.2022-0044 | No match for ‘cyberbully’ or ‘cyber-bully’ in TI/AB/DE/ID |
| 33 | WOS:000669787600001 | Holton, AE Bélair-Gagnon, V Bossio, D Molyneux, L | 2023 | "Not Their Fault, but Their Problem": Organizational Responses to the Online Harassment of Journalists | JOURNALISM PRACTICE | 10.1080/17512786.2021.1946417 | No match for ‘cyberbully’ or ‘cyber-bully’ in TI/AB/DE/ID |
| 34 | WOS:001137186300001 | Lee, NY Park, A | 2024 | Unraveling the Digital Threat: Exploring the Impact of Online Harassment on South Korean Journalists' Professional Roles | JOURNALISM & MASS COMMUNICATION QUARTERLY | 10.1177/10776990231217448 | No match for ‘cyberbully’ or ‘cyber-bully’ in TI/AB/DE/ID |
| 35 | WOS:000520501900001 | Hassan, FM Khalifa, FN El Desouky, ED Salem, MR Ali, MM | 2020 | Cyber violence pattern and related factors: online survey of females in Egypt | EGYPTIAN JOURNAL OF FORENSIC SCIENCES | 10.1186/s41935-020-0180-0 | No match for ‘cyberbully’ or ‘cyber-bully’ in TI/AB/DE/ID |
| 36 | WOS:000978537000001 | Björkenfeldt, O | 2023 | Swedish journalists' perceptions of legal protection against unlawful online harassment | FRONTIERS IN SOCIOLOGY | 10.3389/fsoc.2023.1154495 | No match for ‘cyberbully’ or ‘cyber-bully’ in TI/AB/DE/ID |
| 37 | WOS:000566073700001 | Arafa, A Eshak, ES Abu Salem, E Mohamed, A Mahmoud, O | 2021 | Psychological correlates with violence against women victimization in Egypt | INTERNATIONAL JOURNAL OF MENTAL HEALTH | 10.1080/00207411.2020.1812819 | No match for ‘cyberbully’ or ‘cyber-bully’ in TI/AB/DE/ID |
| 38 | WOS:000468806400008 | Antunovic, D | 2019 | "We wouldn't say it to their faces": online harassment, women sports journalists, and feminism | FEMINIST MEDIA STUDIES | 10.1080/14680777.2018.1446454 | No match for ‘cyberbully’ or ‘cyber-bully’ in TI/AB/DE/ID |
| 39 | WOS:001470201200014 | Yadav, M Dutta, P | 2025 | Navigating Online Hostility: Gendered Trolling of Indian Women Journalists, Legal Interventions, and the Role of Media Literacy | MEDIA EDUCATION-MEDIAOBRAZOVANIE | 10.13187/me.2025.1.138 | No match for ‘cyberbully’ or ‘cyber-bully’ in TI/AB/DE/ID |
| 40 | WOS:000939696200001 | Schoenebeck, S Lampe, C Trieu, P | 2023 | Online Harassment: Assessing Harms and Remedies | SOCIAL MEDIA + SOCIETY | 10.1177/20563051231157297 | No match for ‘cyberbully’ or ‘cyber-bully’ in TI/AB/DE/ID |
| 41 | WOS:001541191800001 | Yasmin, M Jabeen, T Noor, S | 2025 | Examining Digital Hate: Gendered discursive strategies in online harassment of Pakistani influencers | COMPUTERS IN HUMAN BEHAVIOR REPORTS | 10.1016/j.chbr.2025.100759 | No match for ‘cyberbully’ or ‘cyber-bully’ in TI/AB/DE/ID |
| 42 | WOS:000628937400001 | Haslop, C O'Rourke, F Southern, R | 2021 | #NoSnowflakes: The toleration of harassment and an emergent gender-related digital divide, in a UK student online culture | CONVERGENCE-THE INTERNATIONAL JOURNAL OF RESEARCH INTO NEW MEDIA TECHNOLOGIES | 10.1177/1354856521989270 | No match for ‘cyberbully’ or ‘cyber-bully’ in TI/AB/DE/ID |
| 43 | WOS:000376240800005 | Sadowski, H | 2016 | From #aufschrei to hatr.org: digital-material entanglements in the context of German digital feminist activisms | FEMINIST MEDIA STUDIES | 10.1080/14680777.2015.1093090 | No match for ‘cyberbully’ or ‘cyber-bully’ in TI/AB/DE/ID |
| 44 | WOS:001296472300001 | Golbeck, J | 2025 | Misogynistic targeting of women in power predicts broader online harassment patterns | COMPUTATIONAL AND MATHEMATICAL ORGANIZATION THEORY | 10.1007/s10588-024-09387-w | No match for ‘cyberbully’ or ‘cyber-bully’ in TI/AB/DE/ID |
| 45 | WOS:000907162900002 | Einarsdóttir, BB Olafsson, JG | 2022 | The drop hollows out the stone: How politicians and jour- nalists experience harsh discourse and harassment online | ICELANDIC REVIEW OF POLITICS & ADMINISTRATION | 10.13177/irpa.a.2022.18.2.2 | No match for ‘cyberbully’ or ‘cyber-bully’ in TI/AB/DE/ID |
| 46 | WOS:001060346600001 | Park, A Lee, NY | 2024 | Digital Scarlet Letter on Journalists: Weaponized Harassment Against Journalists in South Korea | JOURNALISM PRACTICE | 10.1080/17512786.2023.2250758 | No match for ‘cyberbully’ or ‘cyber-bully’ in TI/AB/DE/ID |
| 47 | WOS:000407751500005 | Munger, K | 2017 | Tweetment Effects on the Tweeted: Experimentally Reducing Racist Harassment | POLITICAL BEHAVIOR | 10.1007/s11109-016-9373-5 | No match for ‘cyberbully’ or ‘cyber-bully’ in TI/AB/DE/ID |
| 48 | WOS:000553425700001 | Haslop, C O'Rourke, F | 2021 | 'I mean, in my opinion, I have it the worst, because I am white. I am male. I am heterosexual': questioning the inclusivity of reconfigured hegemonic masculinities in a UK student online culture | INFORMATION COMMUNICATION & SOCIETY | 10.1080/1369118X.2020.1792531 | No match for ‘cyberbully’ or ‘cyber-bully’ in TI/AB/DE/ID |
| 49 | WOS:000580625300005 | Wegner, J Prommer, E Seidel, CC | 2020 | Free Speech Under Pressure: The Effect of Online Harassment on Literary Writers | MEDIA AND COMMUNICATION | 10.17645/mac.v8i4.3252 | No match for ‘cyberbully’ or ‘cyber-bully’ in TI/AB/DE/ID |
| 50 | WOS:001080130900001 | Inya, O | 2023 | 'This app is evil forest true true': metaphor-based metadiscursive evaluations of Twitter by Nigerians | DIGITAL SCHOLARSHIP IN THE HUMANITIES | 10.1093/llc/fqad063 | No match for ‘cyberbully’ or ‘cyber-bully’ in TI/AB/DE/ID |
| 51 | WOS:000938118700041 | Felmlee, D DellaPosta, D Rodis, PDCI Matthews, SA | 2020 | Can Social Media Anti-abuse Policies Work? A Quasi-experimental Study of Online Sexist and Racist Slurs | SOCIUS | 10.1177/2378023120948711 | No match for ‘cyberbully’ or ‘cyber-bully’ in TI/AB/DE/ID |
| 52 | WOS:000639690500001 | Maddocks, S | 2022 | Feminism, activism and non-consensual pornography: analyzing efforts to end "revenge porn" in the United States | FEMINIST MEDIA STUDIES | 10.1080/14680777.2021.1913434 | No match for ‘cyberbully’ or ‘cyber-bully’ in TI/AB/DE/ID |
| 53 | WOS:001422782100001 | Donevan, M Jonsson, LS Svedin, CG | 2025 | The experience of individuals filmed for pornography production: a history of continuous polyvictimization and ongoing mental health challenges | NORDIC JOURNAL OF PSYCHIATRY | 10.1080/08039488.2025.2464634 | No match for ‘cyberbully’ or ‘cyber-bully’ in TI/AB/DE/ID |
| 54 | WOS:001178021200001 | Perez, J Pepping, CA | 2025 | Relationship experiences of transgender and non-binary adults: exploring dating goals, relationship structures, minority stress, sexual fetishization, and relationship victimization | INTERNATIONAL JOURNAL OF TRANSGENDER HEALTH | 10.1080/26895269.2024.2316690 | No match for ‘cyberbully’ or ‘cyber-bully’ in TI/AB/DE/ID |
| 55 | WOS:000818891500001 | Kempton, SD Connolly-Ahern, C | 2022 | "Who's Going to be a Creep Today?" Understanding the Social Media Experiences of Women Broadcast Journalists | SOCIAL MEDIA + SOCIETY | 10.1177/20563051221108410 | No match for ‘cyberbully’ or ‘cyber-bully’ in TI/AB/DE/ID |
| 56 | WOS:000631223200001 | Kumar, P Gruzd, A Mai, P | 2021 | Mapping out Violence Against Women of Influence on Twitter Using the Cyber-Lifestyle Routine Activity Theory | AMERICAN BEHAVIORAL SCIENTIST | 10.1177/0002764221989777 | No match for ‘cyberbully’ or ‘cyber-bully’ in TI/AB/DE/ID |
| 57 | WOS:000870571900001 | Bhat, P Chadha, K | 2023 | The Mob, the State and Harassment of Journalists via Twitter in India | DIGITAL JOURNALISM | 10.1080/21670811.2022.2134164 | No match for ‘cyberbully’ or ‘cyber-bully’ in TI/AB/DE/ID |
| 58 | WOS:000599228700003 | Bugueño, M Mendoza, M | 2020 | Learning to combine classifiers outputs with the transformer for text classification | INTELLIGENT DATA ANALYSIS | 10.3233/IDA-200007 | No match for ‘cyberbully’ or ‘cyber-bully’ in TI/AB/DE/ID |
| 59 | WOS:000884234200001 | Abhishek, A | 2023 | The State Deputizing Citizens to Discipline Digital News Media: The Case of the IT Rules 2021 in India | DIGITAL JOURNALISM | 10.1080/21670811.2022.2134163 | No match for ‘cyberbully’ or ‘cyber-bully’ in TI/AB/DE/ID |
| 60 | WOS:001406495800001 | Low, E Monsen, J Schow, L Roberts, R Collins, L Johnson, H Hanson, CL Snell, Q Tass, ES | 2025 | Predicting bullying victimization among adolescents using the risk and protective factor framework: a large-scale machine learning approach | BMC PUBLIC HEALTH | 10.1186/s12889-025-21521-0 | No match for ‘cyberbully’ or ‘cyber-bully’ in TI/AB/DE/ID |
| 61 | WOS:000438157700003 | Marwick, AE Caplan, R | 2018 | Drinking male tears: language, the manosphere, and networked harassment | FEMINIST MEDIA STUDIES | 10.1080/14680777.2018.1450568 | No match for ‘cyberbully’ or ‘cyber-bully’ in TI/AB/DE/ID |
| 62 | WOS:000499498800001 | O'Donnell, J | 2020 | Militant meninism: the militaristic discourse of Gamergate and Men's Rights Activism | MEDIA CULTURE & SOCIETY | 10.1177/0163443719876624 | No match for ‘cyberbully’ or ‘cyber-bully’ in TI/AB/DE/ID |
| 63 | WOS:000435499900005 | Kruse, LM Norris, DR Flinchum, JR | 2018 | Social Media as a Public Sphere? Politics on Social Media | SOCIOLOGICAL QUARTERLY | 10.1080/00380253.2017.1383143 | No match for ‘cyberbully’ or ‘cyber-bully’ in TI/AB/DE/ID |
| 64 | WOS:001249013300001 | Wu, L Jamonnak, S Ye, XY Shaw, SL Saxena, N Choi, KS | 2025 | Theoretical and Experimental Framework for Estimating Cyber Victimization Risk in a Hybrid Physical-Virtual World | JOURNAL OF APPLIED SECURITY RESEARCH | 10.1080/19361610.2024.2368969 | No match for ‘cyberbully’ or ‘cyber-bully’ in TI/AB/DE/ID |
| 65 | WOS:000470265300007 | Novoselova, V Jenson, J | 2019 | Authorship and professional digital presence in feminist blogs | FEMINIST MEDIA STUDIES | 10.1080/14680777.2018.1436083 | No match for ‘cyberbully’ or ‘cyber-bully’ in TI/AB/DE/ID |
| 66 | WOS:000668759200001 | Calasanti, A Gerrits, B | 2023 | "You're not my nanny!" Responses to racialized women leaders during COVID-19 | POLITICS GROUPS AND IDENTITIES | 10.1080/21565503.2021.1942084 | No match for ‘cyberbully’ or ‘cyber-bully’ in TI/AB/DE/ID |
| 67 | WOS:000443456100003 | Braithwaite, A | 2016 | It's About Ethics in Games Journalism? Gamergaters and Geek Masculinity | SOCIAL MEDIA + SOCIETY | 10.1177/2056305116672484 | No match for ‘cyberbully’ or ‘cyber-bully’ in TI/AB/DE/ID |
| 68 | WOS:001206462200001 | Sibai, O Luedicke, MK De Valck, K | 2024 | Why Online Consumption Communities Brutalize | JOURNAL OF CONSUMER RESEARCH | 10.1093/jcr/ucae022 | No match for ‘cyberbully’ or ‘cyber-bully’ in TI/AB/DE/ID |
| 69 | WOS:000473463500005 | Kargar, S Rauchfleisch, A | 2019 | State-aligned trolling in Iran and the double-edged affordances of Instagram | NEW MEDIA & SOCIETY | 10.1177/1461444818825133 | No match for ‘cyberbully’ or ‘cyber-bully’ in TI/AB/DE/ID |
| 70 | WOS:000436664800003 | Enarsson, T Naarttijärvi, M | 2016 | Is it all part of the game? Victim differentiation and the normative protection of victims of online antagonism under the European Convention on Human Rights | INTERNATIONAL REVIEW OF VICTIMOLOGY | 10.1177/0269758016628947 | No match for ‘cyberbully’ or ‘cyber-bully’ in TI/AB/DE/ID |
| 71 | WOS:000720665200002 | Are, C | 2021 | Patterns of media coverage repeated in online abuse on high-profile criminal cases | JOURNALISM | 10.1177/1464884919881274 | No match for ‘cyberbully’ or ‘cyber-bully’ in TI/AB/DE/ID |
| 72 | WOS:000736713900001 | Aurpa, TT Sadik, R Ahmed, MS | 2022 | Abusive Bangla comments detection on Facebook using transformer-based deep learning models | SOCIAL NETWORK ANALYSIS AND MINING | 10.1007/s13278-021-00852-x | No match for ‘cyberbully’ or ‘cyber-bully’ in TI/AB/DE/ID |
| 73 | WOS:000876861500001 | Macaraig, A Hameleers, M | 2022 | DefendPressFreedom: Paradigm Repair, Role Perceptions and Filipino Journalists' Counterstrategies to Anti-Media Populism and Delegitimizing Threats | JOURNALISM STUDIES | 10.1080/1461670X.2022.2138949 | No match for ‘cyberbully’ or ‘cyber-bully’ in TI/AB/DE/ID |
| 74 | WOS:000424698900005 | Thompson, L | 2018 | "I can be your Tinder nightmare": Harassment and misogyny in the online sexual marketplace | FEMINISM & PSYCHOLOGY | 10.1177/0959353517720226 | No match for ‘cyberbully’ or ‘cyber-bully’ in TI/AB/DE/ID |
| 75 | WOS:000870700400009 | Pardo, FD | 2022 | Hate speech and freedom of expression in digital environments: social and legal implications | SOLETRAS | 10.12957/soletras.2022.64970 | No match for ‘cyberbully’ or ‘cyber-bully’ in TI/AB/DE/ID |
| 76 | WOS:000333946200005 | Sasson, H Mesch, G | 2014 | Parental mediation, peer norms and risky online behavior among adolescents | COMPUTERS IN HUMAN BEHAVIOR | 10.1016/j.chb.2013.12.025 | No match for ‘cyberbully’ or ‘cyber-bully’ in TI/AB/DE/ID |
| 77 | WOS:000765606900001 | Krieger, MA Santarossa, S Bours, S Woodruff, SJ | 2023 | Men's comments on elite women athletes: cultural narratives around gender and sport on Instagram | FEMINIST MEDIA STUDIES | 10.1080/14680777.2022.2041696 | No match for ‘cyberbully’ or ‘cyber-bully’ in TI/AB/DE/ID |
| 78 | WOS:000531985100001 | Wright, S Trott, V Jones, C | 2020 | 'The pussy ain't worth it, bro': assessing the discourse and structure of MGTOW | INFORMATION COMMUNICATION & SOCIETY | 10.1080/1369118X.2020.1751867 | No match for ‘cyberbully’ or ‘cyber-bully’ in TI/AB/DE/ID |
| 79 | WOS:000647702600009 | Richard, EM Young, SF Walsh, JJ Giumetti, GW | 2020 | Cyberaggression in Work-Related Email: Nomological Network and Links to Victims' Counterproductive Work Behavior | OCCUPATIONAL HEALTH SCIENCE | 10.1007/s41542-020-00056-3 | No match for ‘cyberbully’ or ‘cyber-bully’ in TI/AB/DE/ID |
| 80 | WOS:000427706200003 | Lawson, CE | 2018 | Platform vulnerabilities: harassment and misogynoir in the digital attack on Leslie Jones | INFORMATION COMMUNICATION & SOCIETY | 10.1080/1369118X.2018.1437203 | No match for ‘cyberbully’ or ‘cyber-bully’ in TI/AB/DE/ID |
| 81 | WOS:000443456100012 | Shaw, F | 2016 | "Bitch I Said Hi": The Bye Felipe Campaign and Discursive Activism in Mobile Dating Apps | SOCIAL MEDIA + SOCIETY | 10.1177/2056305116672889 | No match for ‘cyberbully’ or ‘cyber-bully’ in TI/AB/DE/ID |
| 82 | WOS:000405408000010 | Kern, RL Gil-Egui, G | 2017 | Women behaving badly: negative posts on Facebook memorial pages | INFORMATION COMMUNICATION & SOCIETY | 10.1080/1369118X.2016.1257040 | No match for ‘cyberbully’ or ‘cyber-bully’ in TI/AB/DE/ID |
| 83 | WOS:000438157700010 | Alvares, C | 2018 | Online staging of femininity: disciplining through public exposure in Brazilian social media | FEMINIST MEDIA STUDIES | 10.1080/14680777.2018.1447336 | No match for ‘cyberbully’ or ‘cyber-bully’ in TI/AB/DE/ID |
| 84 | WOS:001241761500001 | Rozo, AZ Campo-Archbold, A Díaz-López, D Gray, I Pastor-Galindo, J Nespoli, P Mármol, FG McCoy, D | 2024 | Cyber democracy in the digital age: Characterizing hate networks in the 2022 US midterm elections | INFORMATION FUSION | 10.1016/j.inffus.2024.102459 | No match for ‘cyberbully’ or ‘cyber-bully’ in TI/AB/DE/ID |
| 85 | WOS:001045113800001 | Chua, YT Wilson, L | 2023 | Beyond Black and White: the Intersection of Ideologies in Online Extremist Communities | EUROPEAN JOURNAL ON CRIMINAL POLICY AND RESEARCH | 10.1007/s10610-023-09555-9 | No match for ‘cyberbully’ or ‘cyber-bully’ in TI/AB/DE/ID |
| 86 | WOS:000708225900007 | Park, S Kim, J | 2021 | Tweeting about abusive comments and misogyny in South Korea following the suicide of Sulli, a female K-pop star: Social and semantic network analyses | PROFESIONAL DE LA INFORMACION | 10.3145/epi.2021.sep.05 | No match for ‘cyberbully’ or ‘cyber-bully’ in TI/AB/DE/ID |
| 87 | WOS:001189076500001 | de Roos, MS Veldhuizen-Ochodnicanová, L Hanna, A | 2024 | The Angry Echo Chamber: A Study of Extremist and Emotional Language Changes in Incel Communities Over Time | JOURNAL OF INTERPERSONAL VIOLENCE | 10.1177/08862605241239451 | No match for ‘cyberbully’ or ‘cyber-bully’ in TI/AB/DE/ID |
| 88 | WOS:000372574900028 | Chadaga, AR Villines, D Krikorian, A | 2016 | Bullying in the American Graduate Medical Education System: A National Cross-Sectional Survey | PLOS ONE | 10.1371/journal.pone.0150246 | No match for ‘cyberbully’ or ‘cyber-bully’ in TI/AB/DE/ID |
| 89 | WOS:000715124500018 | Alsawalqa, RO Alrawashdeh, MN Hasan, S | 2021 | Understanding the Man Box: the link between gender socialization and domestic violence in Jordan | HELIYON | 10.1016/j.heliyon.2021.e08264 | No match for ‘cyberbully’ or ‘cyber-bully’ in TI/AB/DE/ID |
| 90 | WOS:001414898700008 | Busón , C Angola, AC Mendoza, KP Rodríguez, AD | 2022 | Analysis of the comments to a presidential pronouncement on COVID-19 in Brazil, made on March 23, 2021 | AMBITOS | 10.12795/Ambitos.2022.i57.08 | No match for ‘cyberbully’ or ‘cyber-bully’ in TI/AB/DE/ID |
| 91 | WOS:000303128600016 | Pujazon-Zazik, MA Manasse, SM Orrell-Valente, JK | 2012 | Adolescents' Self-presentation on a Teen Dating Web Site: A Risk-Content Analysis | JOURNAL OF ADOLESCENT HEALTH | 10.1016/j.jadohealth.2011.11.015 | No match for ‘cyberbully’ or ‘cyber-bully’ in TI/AB/DE/ID |

**Validation note.** The source export contained 345 records; the narrow rule retained 254 records and excluded 91 records. Among the excluded records, 8 contained the spaced form “cyber bullying” in at least one target field and remained excluded because that form was outside the predefined narrow regular expression.
