# Supplementary material for: Language and context in cyberbullying research: bibliometric mapping and conceptual integration of lexical, pragmatic, and media-psychological perspectives (2010–2025)
Source: Front Psychol. 2026 Oct 21;17:1963458. doi: 10.3389/fpsyg.2026.1963458 (PMC13731407; doi:10.3389/fpsyg.2026.1963458)
Supplement: Supplementary file 2 [file Table_2.DOCX]

**Supplementary Table S2. Record-level visibility of selected pragmatic terms across Web of Science metadata fields in the 345-record corpus**

Counts represent the number of bibliographic records in which the specified case-insensitive search pattern occurred at least once within the indicated field. Each record was counted once per field regardless of repeated within-field occurrences. Fields: TI = title; AB = abstract; DE = author keywords; ID = Keywords Plus.

| **Search term** | **Title (TI)** | **Abstract (AB)** | **Author Keywords (DE)** | **Keywords Plus (ID)** |
| --- | --- | --- | --- | --- |
| pragmat* | 2 | 9 | 0 | 0 |
| implicature* | 0 | 0 | 0 | 0 |
| "speech act*" | 0 | 1 | 1 | 0 |
| stance | 2 | 10 | 1 | 0 |
| sarcas* | 5 | 14 | 5 | 0 |
| iron* | 2 | 8 | 3 | 1 |
| politeness | 0 | 1 | 1 | 0 |
| impoliteness | 1 | 3 | 3 | 2 |

**Interpretive note.** Several pragmatic concepts were more visible in titles and abstracts than in indexing fields. For example, stance appeared in 10 abstracts but only one author-keyword record, while sarcasm-related forms appeared in 14 abstracts and five author-keyword records. Implicature-related forms did not occur in any of the four fields.
