# Supplementary material for: Language and context in cyberbullying research: bibliometric mapping and conceptual integration of lexical, pragmatic, and media-psychological perspectives (2010–2025)
Source: Front Psychol. 2026 Oct 21;17:1963458. doi: 10.3389/fpsyg.2026.1963458 (PMC13731407; doi:10.3389/fpsyg.2026.1963458)
Supplement: Supplementary file 3 [file Table_3.DOCX]

**Supplementary Table S3. Illustrative records used for theory-informed interpretation across the four analytical lenses**

Audit basis: the four lens-specific interpretive paragraphs in Section 4.2 of the revised manuscript were cross-checked against the original 345-record Web of Science export using DOI and WoS accession identifiers. A total of 15 unique corpus records were used as illustrative records, producing 16 lens assignments because one record (Marwick and Caplan, 2018) was used under both the lexical-semantic and pragmatics/discourse lenses. Lens-assignment counts were: psychosocial victimization = 1; lexical-semantic harm = 6; pragmatics and discourse = 3; computational detection = 6. External theory, meta-analysis, review, and classifier-bias sources cited in Section 4.2 but not present as records in the 345-record corpus are not counted as illustrative corpus records.

| **Lens** | **Citation** | **Year** | **Article title** | **Source** | **DOI** | **WoS UT** | **Reason for illustrative use** |
| --- | --- | --- | --- | --- | --- | --- | --- |
| Psychosocial victimization | Menesini et al. (2011) | 2011 | The Measurement of Cyberbullying: Dimensional Structure and Relative Item Severity and Discrimination | CYBERPSYCHOLOGY BEHAVIOR AND SOCIAL NETWORKING | 10.1089/cyber.2010.0002 | WOS:000290787800002 | Measurement of perpetrator/victim/bystander roles and cyberbullying severity; used to illustrate the psychosocial measurement tradition. |
| Lexical-semantic harm | Marwick and Caplan (2018) | 2018 | Drinking male tears: language, the manosphere, and networked harassment | FEMINIST MEDIA STUDIES | 10.1080/14680777.2018.1450568 | WOS:000438157700003 | Examines language, misogyny, and networked harassment; used to illustrate harmful lexical and identity-related representations. |
| Lexical-semantic harm | Thompson (2018) | 2018 | "I can be your Tinder nightmare": Harassment and misogyny in the online sexual marketplace | FEMINISM & PSYCHOLOGY | 10.1177/0959353517720226 | WOS:000424698900005 | Examines harassment and misogyny in online interaction; used to illustrate gendered and evaluative harmful language. |
| Lexical-semantic harm | Lawson (2018) | 2018 | Platform vulnerabilities: harassment and misogynoir in the digital attack on Leslie Jones | INFORMATION COMMUNICATION & SOCIETY | 10.1080/1369118X.2018.1437203 | WOS:000427706200003 | Examines harassment and misogynoir on a digital platform; used to illustrate identity-targeted lexical-semantic harm. |
| Lexical-semantic harm | Antunovic (2019) | 2019 | "We wouldn't say it to their faces": online harassment, women sports journalists, and feminism | FEMINIST MEDIA STUDIES | 10.1080/14680777.2018.1446454 | WOS:000468806400008 | Examines online harassment of women sports journalists; used to illustrate gendered harassment discourse and lexical representation. |
| Lexical-semantic harm | Wright et al. (2020) | 2020 | 'The pussy ain't worth it, bro': assessing the discourse and structure of MGTOW | INFORMATION COMMUNICATION & SOCIETY | 10.1080/1369118X.2020.1751867 | WOS:000531985100001 | Analyzes MGTOW discourse and structure; used to illustrate misogynistic labels and discourse-linked lexical harm. |
| Lexical-semantic harm | Holton et al. (2023) | 2023 | "Not Their Fault, but Their Problem": Organizational Responses to the Online Harassment of Journalists | JOURNALISM PRACTICE | 10.1080/17512786.2021.1946417 | WOS:000669787600001 | Examines organizational responses to online harassment of journalists; used to contextualize the institutional consequences of harmful discourse. |
| Pragmatics and discourse | Dynel and Poppi (2019) | 2019 | Risum teneatis, amici?*: The socio-pragmatics of RoastMe humour | JOURNAL OF PRAGMATICS | 10.1016/j.pragma.2018.10.010 | WOS:000457668500001 | Socio-pragmatic analysis of RoastMe humour; used to illustrate context-dependent interpretation, humour, and participant norms. |
| Pragmatics and discourse | Marwick and Caplan (2018) | 2018 | Drinking male tears: language, the manosphere, and networked harassment | FEMINIST MEDIA STUDIES | 10.1080/14680777.2018.1450568 | WOS:000438157700003 | Also used in the pragmatic/discourse lens because interpretation of networked harassment depends on discourse context and identity positioning. |
| Pragmatics and discourse | Zhong et al. (2022) | 2022 | To Be Ethical and Responsible Digital Citizens or Not: A Linguistic Analysis of Cyberbullying on Social Media | FRONTIERS IN PSYCHOLOGY | 10.3389/fpsyg.2022.861823 | WOS:000795593900001 | Linguistic analysis of cyberbullying on social media; used to illustrate context-sensitive lexical and discourse interpretation. |
| Computational detection | Salawu et al. (2020) | 2020 | Approaches to Automated Detection of Cyberbullying: A Survey | IEEE TRANSACTIONS ON AFFECTIVE COMPUTING | 10.1109/TAFFC.2017.2761757 | WOS:000619246800001 | Survey of automated cyberbullying detection; used to represent the development and limitations of computational approaches. |
| Computational detection | Lu et al. (2020) | 2020 | Cyberbullying detection in social media text based on character-level convolutional neural network with shortcuts | CONCURRENCY AND COMPUTATION-PRACTICE & EXPERIENCE | 10.1002/cpe.5627 | WOS:000505291800001 | Character-level convolutional neural-network detection of cyberbullying; used to illustrate deep-learning feature extraction. |
| Computational detection | Dewani et al. (2021) | 2021 | Cyberbullying detection: advanced preprocessing techniques & deep learning architecture for Roman Urdu data | JOURNAL OF BIG DATA | 10.1186/s40537-021-00550-7 | WOS:000733902200001 | Roman Urdu cyberbullying detection using preprocessing and deep learning; used to illustrate computational work in a lower-resource language. |
| Computational detection | Chia et al. (2021) | 2021 | Machine Learning and feature engineering-based study into sarcasm and irony classification with application to cyberbullying detection | INFORMATION PROCESSING & MANAGEMENT | 10.1016/j.ipm.2021.102600 | WOS:000658372100038 | Machine-learning study of sarcasm and irony with application to cyberbullying detection; used to illustrate computational handling of pragmatic cues. |
| Computational detection | Iwendi et al. (2023) | 2023 | Cyberbullying detection solutions based on deep learning architectures | MULTIMEDIA SYSTEMS | 10.1007/s00530-020-00701-5 | WOS:000578902800002 | Deep-learning architectures for cyberbullying detection; used to illustrate the prominence of neural detection approaches. |
| Computational detection | Mazari et al. (2024) | 2024 | BERT-based ensemble learning for multi-aspect hate speech detection | CLUSTER COMPUTING-THE JOURNAL OF NETWORKS SOFTWARE TOOLS AND APPLICATIONS | 10.1007/s10586-022-03956-x | WOS:000907162100003 | BERT-based ensemble learning for multi-aspect hate-speech detection; used to illustrate transformer-based adjacent harmful-language detection. |

**External sources cited in Section 4.2 but not counted as illustrative corpus records.** Chen et al. (2025), Lo Cricchio et al. (2021), Zhao and Yu (2021), Kowalski et al. (2014), Harris et al. (2022), and Rhee (2023) were used as external interpretive/contextual literature rather than as records selected from the mapped 345-record corpus.
